# Identification of Candidate Forage Yield Genes in Sorghum (*Sorghum bicolor* L.) Using Integrated Genome-Wide Association Studies and RNA-Seq

**DOI:** 10.3389/fpls.2021.788433

**Published:** 2022-01-11

**Authors:** Lihua Wang, Yanlong Liu, Li Gao, Xiaocui Yang, Xu Zhang, Shaoping Xie, Meng Chen, Yi-Hong Wang, Jieqin Li, Yixin Shen

**Affiliations:** ^1^College of Agro-Grassland Science, Nanjing Agricultural University, Nanjing, China; ^2^College of Agriculture, Anhui Science and Technology University, Fengyang, China; ^3^Department of Biology, University of Louisiana at Lafayette, Lafayette, LA, United States

**Keywords:** sorghum, forage yield trait, genome-wide association study, RNA-seq, candidate gene

## Abstract

Genetic dissection of forage yield traits is critical to the development of sorghum as a forage crop. In the present study, association mapping was performed with 85,585 SNP markers on four forage yield traits, namely plant height (PH), tiller number (TN), stem diameter (SD), and fresh weight per plant (FW) among 245 sorghum accessions evaluated in four environments. A total of 338 SNPs or quantitative trait nucleotides (QTNs) were associated with the four traits, and 21 of these QTNs were detected in at least two environments, including four QTNs for PH, ten for TN, six for SD, and one for FW. To identify candidate genes, dynamic transcriptome expression profiling was performed at four stages of sorghum development. One hundred and six differentially expressed genes (DEGs) that were enriched in hormone signal transduction pathways were found in all stages. Weighted gene correlation network analysis for PH and SD indicated that eight modules were significantly correlated with PH and that three modules were significantly correlated with SD. The blue module had the highest positive correlation with PH and SD, and the turquoise module had the highest negative correlation with PH and SD. Eight candidate genes were identified through the integration of genome-wide association studies (GWAS) and RNA sequencing. Sobic.004G143900, an indole-3-glycerol phosphate synthase gene that is involved in indoleacetic acid biosynthesis, was down-regulated as sorghum plants grew in height and was identified in the blue module, and Sobic.003G375100, an SD candidate gene, encoded a DNA repair RAD52-like protein 1 that plays a critical role in DNA repair-linked cell cycle progression. These findings demonstrate that the integrative analysis of omics data is a promising approach to identify candidate genes for complex traits.

## Introduction

Sorghum is an important grain and forage crop. It is widely cultivated worldwide because of its broad adaptability and tolerance to drought, waterlogging, and salinity (Rooney et al., 2007). In addition, cultivation of forage sorghum has recently increased to meet the demand of growing domestic animal production industries, especially in arid and semi-arid regions with perennial water shortages (Huang et al., 2020). Genetic dissection of sorghum yield traits will facilitate the development of sorghum as a high-yielding forage crop that can be used for animal production.

Improvement of forage yield has been a major objective of forage sorghum breeding. Since forage yield traits are usually controlled by many genes, genome-wide association studies (GWAS), which are useful for dissecting complex traits, have been used extensively to map forage yield-related traits in sorghum. The majority of these studies have been cataloged in the Sorghum QTL Atlas (Mace et al., 2019).^1^ In fact, as of September 7, 2021, the database included 61 quantitative trait loci (QTLs) for total dry biomass from eight studies, 67 QTLs for fresh biomass from 10 studies, 413 QTLs for plant height from 48 studies, 168 QTLs for tiller number from 18 studies, and 37 QTLs for stem diameter from six studies. In addition, Spindel et al. (2018) reported another 213 genomic regions that are associated with sorghum biomass and/or drought tolerance, and Habyarimana et al. (2020) reported 42 single-nucleotide polymorphisms (SNPs) associated with plant height, eight with dry mass fraction of fresh material, and 17 with dry biomass yield in sorghum. Kong et al. (2020) reported six QTLs that were related to basal stem diameter, six to middle stem diameter, and five to rachis diameter explained 28.9, 26.0, and 20.0% of phenotypic variation for the corresponding traits, respectively. Dos Santos et al. (2020) used 100,435 SNP markers to identify associations between sorghum plant height and dry forage yield and reported that early season plant height could be used to select for dry forage yield. Chen et al. (2020) identified a *biomass yield 1* (*by1*) mutant that affected sorghum biomass and grain yield through primary and secondary metabolism regulation *via* the shikimate pathway.

RNA sequencing (RNA-Seq) can be used to characterize or identify genes, as well as to obtain precise measurements of transcript levels (Wang et al., 2009). It is also a valuable tool for dissecting gene regulation networks (Marguerat and Bähler, 2010) *via* identification of differentially expressed genes (DEGs). Studies also show that the combination of RNA-Seq and GWAS can be used to narrow down candidate genes at specific QTLs. For example, Yan et al. (2020) combined GWAS and RNA-Seq to identify five candidate genes underpinning ketosis in cattle, and Zhang et al. (2021) mapped 178 peanut seed composition-associated QTLs with GWAS and used RNA-Seq analysis to identify 282 QTL-associated DEGs, including 16 candidate genes for seed fatty acid metabolism and protein synthesis.

In this study, we carried out GWAS analysis for plant height (PH), tiller number (TN), stem diameter (SD), and fresh weight per plant (FW) for 245 sorghum accessions grown across four environments (two locations × 2 years). Dynamic transcriptome expression profiling was performed at four development stages to identify QTL-related DEGs. The results of this integrated approach will improve the current understanding of the genetic mechanisms underlying forage sorghum yield.

## Materials and Methods

### Plant Materials and Trait Measurement

The 245 sorghum accessions were used for forage quality characters as described previously (Li et al., 2018). The sorghum accessions were grown in four environments (2 locations × 2 years), i.e., Fengyang campus of Anhui Province (Fengyang, China, 32°52′N, 177°33′E) in 2015 and 2016, and Tengqiao town of Hainan Province (Tengqiao, China, 18°24′N, 109°45′E) in 2016 and 2017. All experiments were performed using a completely randomized block design with three replicates each. Sorghum cultivar Tx430 was used to perform RNA-seq. It was grown at Fengyang campus.

The four yield traits (PH, TN, SD, and FW) were measured when all accessions were at the heading stage. The middle stem of each plant was used to measure SD, and only aerial plant parts were used to determine FW.

### DNA Extraction, Sequencing, and Single-Nucleotide Polymorphism Analysis

Total DNA was extracted using a DNAsecure Plant Kit (Cat. No. DP320, Qiagen, Hilden, N.W, Germany). Library construction, restriction site-associated DNA (RAD) sequencing, and SNP analysis were performed as described previously (Li et al., 2018).

### Population Structure Analysis

Linkage disequilibrium (LD) analysis was performed using PopLDdecay, with a MaxDist of 1,000 kb. All SNPs were filtered for population structure (Q), and relative kinship analysis (K) was performed using Plink v1.07 (MAF < 0.05, *r*^2^ = 0.2; Purcell et al., 2007). Number of clusters in the population (*k*) was set from 1 to 10, with five independent runs (Pritchard et al., 2000).

### Genome-Wide Association Study

GWAS was performed using TASSEL 5.2.70 (Bradbury et al., 2007), with a mixed linear model (MLM) to calculate associations and the incorporation of Q matrix/PCA and kinship data (K; Zhao et al., 2011). The MLM was applied using default settings (P3D for variance component analysis and compression set to the optimum level). For MLM (Q + K), the significance threshold for significantly associated markers was set to *p* ≤ 4.06 × 10^–4^ or [-log10 (*p*) = 3.39], as described previously (Li et al., 2018).

### RNA-Seq and Data Analysis

Four weeks after planting, Tx430 leaves were sampled every 2 weeks until 10 weeks after planting, representing stages 1–4, respectively. The samples were flash-frozen in liquid nitrogen and stored at –80°C before RNA extraction. Each sample had three biological replicates. Total RNA was extracted using an RNAprep Pure Plant Kit (Tiangen, Beijing, China). Gel electrophoresis and a BioDrop (Biochrom, Cambridge, London, United Kingdom) were used to measure the quality and quantity of total RNA. Libraries were constructed and sequenced at the Beijing Genomics Institute.

Raw data were initially filtered using SOAPnuk v1.5.2 (Chen et al., 2018), and then histat2 (Kim et al., 2019) was used to map clean reads to a sorghum reference genome of BTx623 version 3.1.1 (McCormick et al., 2018).^2^ Differential expressed genes (DEGs) were identified using R package DESeq2 with a padj < 0.05-Benjamini-Hochberg multiple test correction (FDR) and the absolute value of a log2 (FC) > 1 (Love et al., 2014). Both gene Ontology (GO) and Kyoto Encyclopedia of Genes and Genomes (KEGG) enrichment analyses were performed using the “clusterProfiler” package in R (Wu et al., 2021). The enrichment results of GO and KEGG pathways were obtained using *P* < 0.05 as the significance threshold. The top 5 KEGG pathways and top 5 terms of each GO domain were identified.

Weighted gene correlation network analysis (WGCNA) was performed using the R package “WGCNA” (Langfelder and Horvath, 2008). Firstly, the genes were ranked by median absolute deviation from large to small, and the top 50% genes were selected for WGCNA using the “goodSamplesGenes” function in package “WGCNA.” Subsequently, the power parameter ranging from 1 to 20 was screened out using the “pickSoftThreshold” function in package “WGCNA.” A suitable soft threshold of 8 was selected, as it met the degree of independence of 0.85 with the minimum power value. Finally, modules were obtained following dynamic branch cutting with a merging threshold of 0.25. The modules were visualized by the “plotDendroAndColors” function in package “WGCNA.” The correlation map between modules and traits was visualized using the R package “ggcor.”

### Candidate Gene Mining and Data Analysis

Stable QTLs were those detected across two environments. Genes within 50kb of QTL-associated SNPs were considered for further analysis based on the LD results. The candidate genes in the QTLs were obtained according to the reference genome (*Sorghum bicolor* v3.1.1, McCormick et al., 2018) and annotation GFF3 file (*Sorghum bicolor* v3.1.1)^3^ using BEDTools (Quinlan and Hall, 2010).

The phenotypic mean data of PH, TN, SD, and FW were calculated with “Descriptive Statistics” pack in “Data Analysis” tool using Excel 2010. Correlation analysis and histogram construction were performed for the traits using the “PerformanceAnalytics” package in R.

## Results

### Phenotypic Variation Among Accessions

Extensive variation in PH, TN, SD, and FW was observed in all four environments in the 245 accessions (Table 1). The extent of variation for the traits ranged from 1.5- to 5.6-fold. PH ranged from 90.0 to 476.7 cm, with 3.3–5.3-fold variation in the different environments, whereas TN ranged from 0 to 6.3, with 3.5–5.6-fold variation, SD ranged from 5.10 to 29.55 mm, with 1.5–1.8-fold variation, and FW ranged from 0.073 to 5.830 kg, with 2.4–4.9- fold. Mean PH, SD, and FW were significantly lower at Tengqiao (Tq) than at Fengyang (Fy), but no significant difference was observed for TN. It suggests that PH, SD, and FW, but not TN, were affected by photoperiod which was shorter at Tengqiao than at Fengyang.

**TABLE 1:** Statistical descriptions of four yield-related traits in the 245 sorghum accessions evaluated in four environments.

Trait-environment*	Min	Max	Mean ± *SD*
PH-2015Fy	91	470	310.0 ± 59.65
PH-2016Tq	90	385.8	223.4 ± 51.35
PH-2016Fy	90.3	476.7	321.6 ± 65.98
PH-2017Tq	102.7	343	202.3 ± 43.53
TN-2015Fy	0	6.3	1.15 ± 1.25
TN-2016Tq	0	6.3	1.77 ± 1.34
TN-2016Fy	0	6.0	1.07 ± 1.12
TN-2017Tq	0	5.0	0.94 ± 1.11
SD-2015Fy	8.34	29.55	18.59 ± 3.49
SD-2016Tq	6.58	20.19	11.42 ± 2.17
SD-2016Fy	6.40	26.80	17.41 ± 3.10
SD-2017Tq	5.10	20.90	11.36 ± 2.25
FW-2015Fy	0.328	2.897	1.137 ± 0.687
FW-2016Tq	0.160	1.030	0.421 ± 0.164
FW-2016Fy	0.073	5.830	1.182 ± 0.659
FW-2017Tq	0.077	1.587	0.323 ± 0.128

*The four traits included plant height (PH), tiller number (TN), stem diameter (SD), and fresh weight per plant (FW). Fengyang (Fy) and Tengqiao (Tq) were the two locations used for field evaluation of the traits.

**FIGURE 1:**
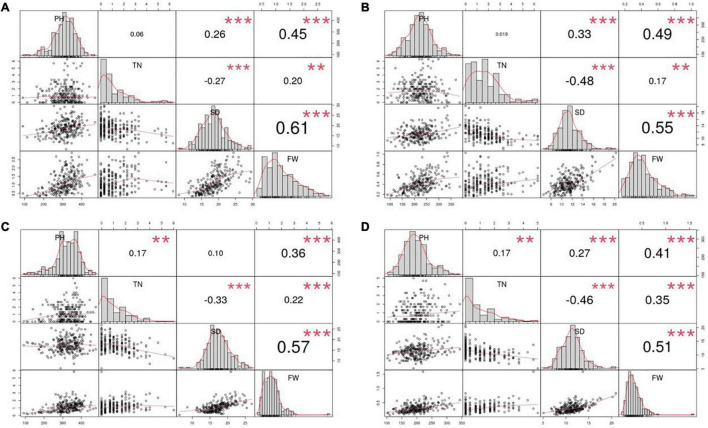
Pearson correlation coefficients for the four yield traits evaluated in the four environments. Four environments: **(A)** 2015Fy, **(B)** 2016Tq, **(C)** 2016Fy, and **(D)** 2017Tq. Four traits: Plant height (PH), tiller number (TN), stem diameter (SD), and fresh weight per plant (FW). ** Indicates significance level at 0.01. *** Indicates significance level at 0.001.

Furthermore, PH, FW, and SD were normally distributed, whereas the distribution of TN was relatively skewed in all four environments. According to Pearson’s correlation coefficients, FW was significantly and positively correlated with PH, SD and TN in all four environments, whereas TN was significantly and negatively correlated with SD (Figure 1). It suggests that the traits are genetically linked or that the traits are affected by genes with pleiotropic effects.

### Linkage Disequilibrium

**FIGURE 2:**
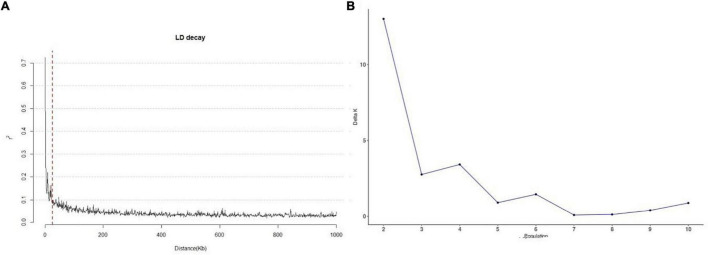
Linkage disequilibrium decay and the population structure of 245 sorghum accessions. **(A)** Genome distribution of *r*^2^-values estimated from 245 sorghum accessions. The red dotted line represents 25 kb. **(B)** Calculation of △*K* based on the value of Ln P(D) between successive *K*-values.

LD in the 245 accessions was calculated using parameter *r*^2^ with 3,026 SNPs. The LD in the 245 accessions decayed after 25 kb on average (Figure 2A), which suggests that the QTLs detected in multiple environments were less than 25 kb from the causal marker(s). The best *K*-value in the population structure was 7 (Figure 2B) and was used for GWAS analysis.

### Genome-Wide Association Analysis

GWAS was performed using a MLM and 85,585 SNP markers. A total of 338 SNPs, or quantitative trait nucleotides (QTNs), were associated with the four yield traits with phenotypic variation explained (PVE) ranged from 4.1 to 57.07% (Supplementary Table 1). Seventy-four SNPs were associated with PH, and 97, 83, and 84 were associated with TN, SD and FW. The association *p*-values for the QTNs ranged from 9.38E-04 to 7.90E-12. The QTNs were distributed relatively evenly across the 10 chromosomes with the highest of 38 QTNs on chromosome 3 and the lowest of 29 on chromosome 6. The numbers of QTNs detected in each of the four environments were not significantly different: 90 QTNs in 2015Fy, 85 in 2016Tq, 87 in 2016Fy, and 76 QTNs in 2017Tq.

In addition, 21 stable QTNs were detected in two environments at least. Four PH QTNs were detected on chromosomes 1, 3, 4, and 8 (one each) in two environments. Ten TN QTNs were detected on chromosomes 3, 4, 5, 7, and 10 in all four environments. Six SD QTNs were detected on chromosomes 3, 7, 8, and 10. One FW QTN was detected on chromosome 8. In these stable QTNs, the PVE of eight QTNs was greater than 10% in both environments, and the PVE of S4_1261758 and S3_69018585 were greater than 20% (Table 2).

**TABLE 2:** Quantitative trait nucleotides (QTNs) associated with four forage yield traits across two or more environments.

Trait	QTNs	Chromosome	Position	P1/P2*	PVE1/PVE2*
**PH**	PH_S1_46978339	1	46,978,339	2.20E-07/8.71E-05	10.98%/7.01%
	PH_S3_4829992	3	4,829,992	1.96E-05/6.20E-04	7.57%/5.39%
	PH_S4_43509891	4	43,509,891	8.73E-05/4.36E-04	6.89%5.53%
	PH_S8_53045404	8	53,045,404	6.20E-06/5.31E-04	8.25%/4.85%
**TN**	TN_S3_6239628	3	6,239,628	2.36E-06/1.68E-05	20.82%14.13%
	TN_S3_68669720	3	68,669,720	7.98E-07/5.46E-08	7.36%/7.64%
	TN_S4_1261758	4	1,261,758	5.07E-06/2.50E-07	35.73%/23.39%
	TN_S5_4584927	5	4,584,927	9.11E-05/4.04E-04	13.84%/16.59%
	TN_S7_58337639	7	58,337,639	3.74E-05/5.77E-05	9.59%/11.44%
	TN_S8_40603220	8	40,603,220	8.90E-06/3.71E-06	23.29%/14.02%
	TN_S9_4170299	9	4,170,299	3.70E-05/6.50E-05	18.21%/22.21%
	TN_S9_19908507	9	19,908,507	4.33E-06/5.40E-05	7.42%/6.85%
	TN_S10_4454931	10	4,454,931	1.47E-07/4.56E-06	32.63%/25.77%
	TN_S10_51545993	10	51,545,993	4.14E-05/4.40E-05	8.38%/7.53%
**SD**	SD_S3_4337503	3	4,337,503	4.65E-06/7.62E-08	10.21%/13.17%
	SD_S3_66578380	3	66,578,380	1.03E-05/6.51E-06	8.24%9.08%
	SD_S3_69018585	3	69,018,585	1.95E-05/1.38E-05	26.58%22.95%
	SD_S7_55857920	7	55,857,920	2.22E-05/2.23E-05	9.97%/10.87%
	SD_S8_55262749	8	55,262,749	8.73E-06/5.50E-06	9.89%9.50%
	SD_S10_54212511	10	54,212,511	1.67E-04/3.35E-07	7.32%12.07%
**FW**	FW_S8_11923031	8	11,923,031	1.73E-04/5.42E-04	5.79%/5.15%

*P1 and PVE1 represent p-value and PVE in one environment, respectively, and P2 and PVE2 represent the p-value and PVE in another environment. PVE, phenotypic variation explained.

### RNA-Seq in the Four Growth Stages

**FIGURE 3:**
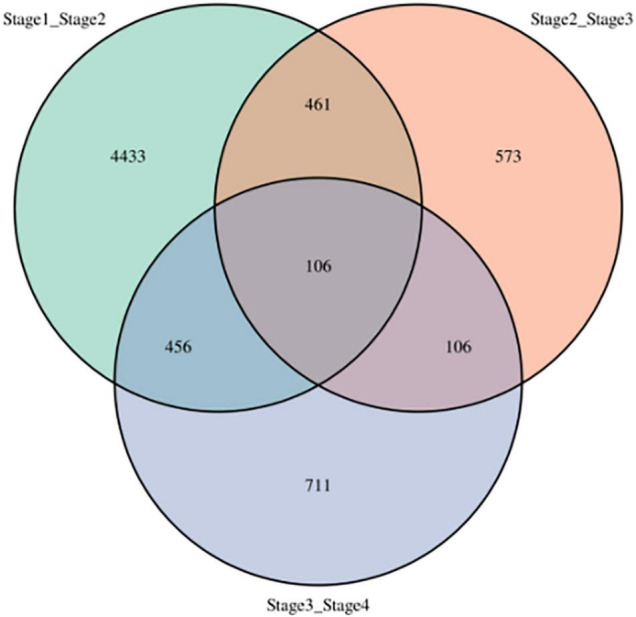
Differentially expressed genes at four development stages in sorghum. stage1_stage2: stages 1 and 2, stage2_stage3: stages 2 and 3, stage3_stage4: stages 3 and 4.

Number of DEGs decreased with development stage from 5,456 (2,417 up- and 3,039 down-regulated) between stages 1 and 2 (stage1_stage2) to 1,246 (684 up- and 562 down-regulated) between stages 2 and stage 3 (stage2_stage3), and 1,379 (543 up- and 836 down-regulated) between stages 3 and stage 4 (stage3_stage4, Figure 3). In addition, the three sets of DEGs included 4,443, 573, and 711 unique DEGs, respectively. However, 106 DEGs were shared among all three-stage comparisons, which suggested that the DEGs play important roles in vegetative development.

**FIGURE 4:**
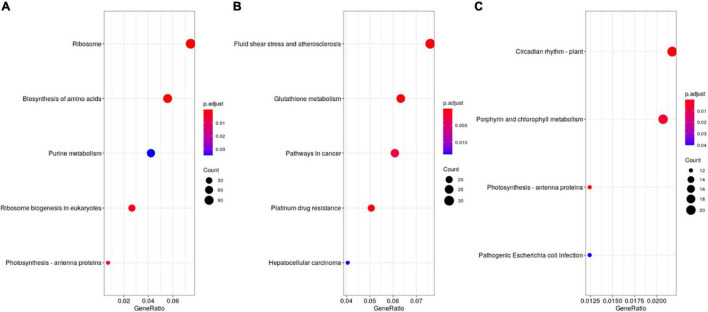
KEGG enrichment of differentially expressed genes in the four development stages of sorghum. **(A)** stage1_stage2. **(B)** stage2_stage3. **(C)** stage3_stage4.

Furthermore, KEGG enrichment analysis indicated that the stage1_stage2, stage2_stage3, and stage3_stage4 DEGs were associated with pathways related to ribosome and amino acid biosynthesis, stress and glutathione metabolism, and circadian rhythm and photosynthesis, respectively (Figure 4). From stage3 to stage4, sorghum transitioned from vegetative to reproductive development. Four *Flowering Locus T-like* (*FTL*) genes were significantly up-regulated at stage 4, and the expression of two phytochrome biosynthesis-related genes were also affected in the stage3_stage4 DEGs (Table 3). The *FTL* genes may play a role in the transition between vegetative to reproductive stages. The 106 DEGs shared among the four developmental stages encoded proteins involved in plant hormone signal transduction, including three jasmonate-zim-domain proteins that were down-regulated in stage1_stage2 and stage3_stage4 but up-regulated in stage2_stage3 (Table 3). In *Arabidopsis*, JAZ10/JAZ11 regulates root growth (Liu et al., 2021).

**TABLE 3:** Circadian rhythm- and hormone signal transduction-related enriched genes.

Pathway	Gene number	Comparison (Log2 fold change)			Function annotation
		Stage 1_ stage2	Stage2_stage3	Stage3_stage4	
Circadian rhythm	Sobic.003G017200	NA	3.24 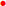	5.67 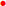	FTL1
	Sobic.006G128500	–2.94 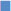	NA	3.00 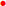	FTL6
	Sobic.010G045100	–2.94 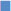	NA	3.00 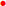	FTL2
	Sobic.002G262500	NA	NA	2.25 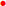	FTL4
	Sobic.001G087100	NA	NA	–1.35 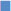	Phytochrome C
	Sobic.004G312600	1.44 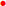	NA	1.32 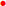	COP1
	Sobic.009G238000	1.32 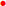	NA	1.17 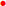	Suppressor of phytochrome A
Hormone signal transduction	Sobic.001G259900	–6.35 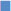	6.87 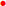	–8.13 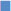	Jasmonate-zim-domain protein 1
	Sobic.006G056400	–5.15 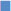	4.10 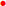	–2.39 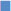	Jasmonate-zim-domain protein 10 (JAZ10)
	Sobic.001G259600	–3.73 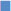	4.70 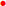	–5.04 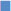	Jasmonate-zim-domain protein 11 (JAZ11)

*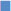, down-regulated; 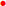, up-regulated.*

**FIGURE 5:**
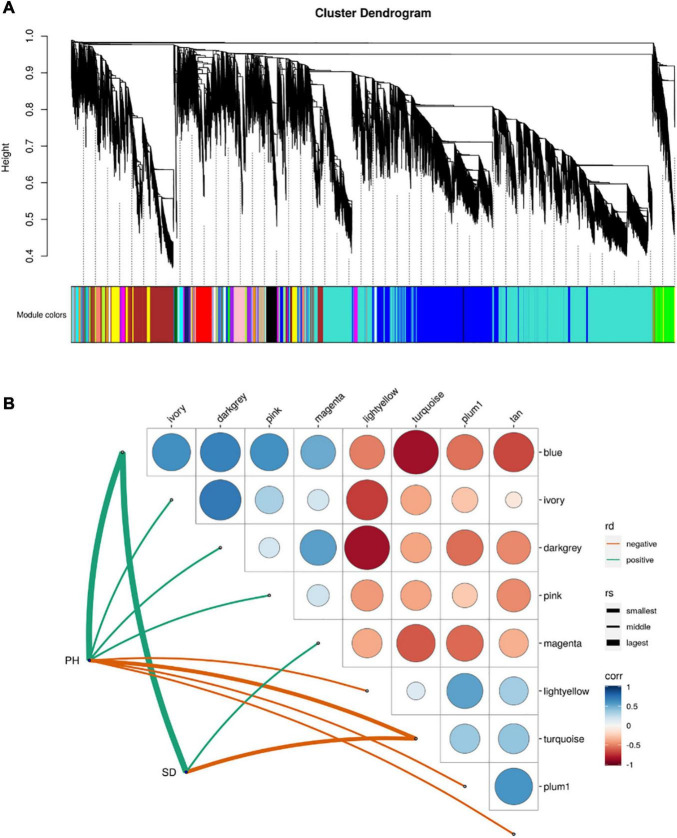
Module-trait relationship from weighted gene correlation network analysis. **(A)** Module-trait map. **(B)** Significant correlation map between traits and modules. rd-represents direction of correlation; rv-represents size of *r*-value.

Weighted gene correlation network analysis (WGCNA) indicated that 45 separate modules were correlated with PH and SD (Figure 5A and Supplementary Table 4). Eight of the modules were significantly correlated with PH, and three with SD (*p* < 0.05; Figure 5B). The blue module had the highest positive correlation with PH and SD (*r* = 0.914 and *r* = 0.915, respectively), and the turquoise module had the highest negative correlation with PH and SD (*r* = –0.717 and *r* = –0.958, respectively). There were 4,733 and 8,635 genes in the blue and turquoise modules, respectively, which indicates that the development of PH and SD is complex and involves a large number of genes.

The DEGs in the blue module were up-regulated in all three-stage comparisons, whereas the most of DEGs in the turquoise module were down-regulated in the stage1_stage2 and stage3_stage4 (Supplementary Table 5). KEGG enrichment analysis indicated that the genes in the blue module were mainly enriched in pathways related to spliceosome and protein processing in the endoplasmic reticulum (Supplementary Figure 1A), whereas the genes in the turquoise module were mainly enriched in ribosome and purine metabolism pathways (Supplementary Figure 1B).

### Candidate Genes by Genome-Wide Association Studies and RNA-Seq

The sorghum annotation file was used to annotate genes associated with the 21 stable QTNs. Of the 86 candidate genes associated with the 21 stable QTNs (Supplementary Table 2), PH, TN, and SD were associated with 14, 40, and 32, respectively.

Further analysis reduced the number of candidate genes to eight that were associated with seven QTNs by RNA-seq (Table 4). A gene for indole-3-glycerol phosphate synthase (Zhao, 2010), which is involved in the biosynthesis of indole-3-acetic acid (IAA), was associated with PH and was down-regulated in the stage1_stage2 comparison. Of the four candidate genes associated with TN, two were up-regulated, and two were down-regulated. Of the three candidate genes associated with SD, two (*Sobic.003G047700* and *Sobic.003G047800*) encoded cytokinin-O-glucosyltransferase 3 and both genes were up-regulated. Another (*Sobic.003G375100*) encoded a mitochondrial DNA repair RAD52-like protein 1, which plays a very important role in plant development, especially vegetative development (Table 4).

**TABLE 4:** Candidate yield-related genes in forage sorghum.

No.	Trait	Chr	Start	End	Gene number	Gene name	DEGs	Module
1	PH	Chr04	43,484,891	43,534,891	Sobic.004G143900	Indole-3-glycerol phosphate synthase	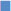	Turquoise
2	TN	Chr03	68,644,720	68,694,720	Sobic.003G370700	ZOS1-18—C2H2 zinc finger protein	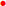	Blue
3	TN	Chr04	1,236,758	1,286,758	Sobic.004G015600	NA	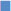	Turquoise
4	TN	Chr07	58,312,639	58,362,639	Sobic.007G151400	Cytokinin dehydrogenase precursor	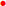	Blue
5	TN	Chr10	4,429,931	4,479,931	Sobic.010G057300	Glycosyltransferase	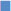	Turquoise
6	SD	Chr03	4,312,503	4,362,503	Sobic.003G047700 Sobic.003G047800	Cytokinin-O-glucosyltransferase 3 Cytokinin-O-glucosyltransferase 3	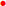 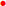	Blue Blue
7	SD	Chr03	68,993,585	69,043,585	Sobic.003G375100	DNA repair RAD52-like protein 1, mitochondrial	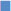	Turquoise

*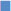, down-regulated; 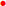, up-regulated.*

## Discussion

GWAS represent an important approach for dissecting the genetic architecture of complex traits in plants (Aulchenko et al., 2007; Liu and Yan, 2019). However, the approach is limited by its high rate of false positives (Cortes et al., 2021). In the future, development in GWAS methodology (Zhu et al., 2008) and multi-environment analysis (Hall et al., 2010) will minimize the rate of false positives. In the present study, MLM and multiple testing environments were used to perform GWAS for four forage sorghum yield traits. Among the 338 QTNs identified, 21 were detected in at least two environments. Thus, the use of multiple testing environments significantly reduced the number of candidate QTNs.

To evaluate linkage strength, the 21 QTNs were compared to QTNs from other studies curated in the Sorghum QTL Atlas (Mace et al., 2019). The comparison identified 12 QTNs (two for PH, six for TN and four for SD) that overlapped with previously published QTLs (Supplementary Table 3). However, no overlapping QTNs were identified for the FW QTNs, probably because of the low heritability of forage yield in sorghum (Shiringani and Friedt, 2011). This observation may also explain why only a single FW QTN was detected in more than one environment. Previous studies have suggested that plant height can be used for indirect selection of forage yield (Fernandes et al., 2018; Dos Santos et al., 2020; Habyarimana et al., 2020). In the present study, 12 of 21 QTNs were identified that overlapped with QTLs from previous studies. These QTNs may provide a robust tool for gene cloning and breeding.

Plant height was also strongly correlated with forage yield in the present study. One of the candidate PH genes encodes indole-3-glycerol phosphate synthase. The gene (Sobic.004G143900) was down-regulated in the stage1_stage2 comparison during sorghum vegetative development. According to RNA-Seq data available in Phytozome 13 (Goodstein et al., 2012; McCormick et al., 2018; see text footnote 2) the expression of the gene is highest in young stems (85.252, stem 1 cm vegetative) and decreases with plant development (15.983, stem mid internode.anthesis). The phytohormone IAA plays a vital role in plant growth (Zhao, 2010) and indole-3-glycerol phosphate synthase serves as a branchpoint compound in the Trp-independent IAA *de novo* biosynthetic pathway (Ouyang et al., 2000). Sun et al. (2020) reported that YABBY2b controls plant height by regulating indole-3-acetic acid-amido synthetase expression in tomato and demonstrated that silencing the indole-3-acetic acid-amido synthetase gene increased plant height. As mentioned above, the indole-3-glycerol phosphate synthase gene Sobic.004G143900 was also down-regulated in the stage1_stage2 comparison.

It is intriguing that the DNA repair *RAD52* gene Sobic.003G375100 was associated with SD in the present study. First, the initial growth of sorghum stem occurs primarily through an increase in cell number (Kebrom et al., 2017), which is achieved *via* mitosis. In *Saccharomyces cerevisiae*, Rad52 participates in the homologous recombination pathway for repairing double-strand DNA breaks, by seeking out and mediating the annealing of homologous DNA strands. Once double-strand DNA breaks are induced, Rad52 relocalizes from a diffuse nuclear distribution to distinct foci, almost exclusively during the S phase of mitosis, thereby demonstrating coordination between recombination repair and DNA replication (Lisby et al., 2001). In mammalian cells, RAD52 plays a similar role in DNA strand exchange and annealing during homologous recombination. In mouse bronchial epithelial cells, *Rad52* blockade slows cell growth and induces senescence, whereas the overexpression of *Rad52* accelerates cell proliferation (Lieberman et al., 2016). Therefore, whether this gene drives SD in sorghum needs further investigation.

RNA-Seq is an important tool for studying gene expression in the whole genome (Tai et al., 2016). However, it is difficult to identify potentially key genes because RNA-Seq usually yields a large number of DEGs (Zhang et al., 2020). In the present study, the blue and turquoise modules had strong correlations with PH and SD, respectively, even though the two modules contained thousands of genes. These indicate that the development of PH and SD are complex and that the large number of genes also hinders the identification of candidate genes for traits of interest.

Even though the application of GWAS to identify candidate genes for important traits is hindered by the high rate of false positives (Xie et al., 2019), the approach can be improved through integration with RNA-Seq. Recent studies have demonstrated the feasibility of this integrated approach in both animals and plants (Xie et al., 2019; Yan et al., 2020). In the present study, the integration of GWAS with RNA-Seq significantly reduced the number of candidate genes responsible for PH, TN, and SD. Further investigation of two of the candidate genes, Sobic.004G143900 and Sobic.003G375100, may provide valuable insight into the molecular mechanisms underlying PH and SD in sorghum. The present study demonstrates the usefulness of the integrative analysis of omics data for identifying candidate genes that underlie complex traits as well as genes for future transgenic studies.

## Data Availability Statement

The datasets presented in this study can be found in online repositories. The names of the repository/repositories and accession number(s) can be found below: NCBI SRA BioProject, accession no: PRJNA780207.

## Author Contributions

LW, YL, LG, XY, XZ, SX, and MC phenotyped plant height (PH), tiller number (TN), stem diameter (SD), and fresh weight for per plant (FW) in the four environments at Fengyang and Tengqiao. LW analyzed GWAS results. JL performed GWAS and LD analysis. JL and Y-HW analyzed RNA-seq results and revised the manuscript. YS took part in the planning of the experiments and revised the manuscript. All authors have read and approved the manuscript for publication.

## Conflict of Interest

The authors declare that the research was conducted in the absence of any commercial or financial relationships that could be construed as a potential conflict of interest.

## Publisher’s Note

All claims expressed in this article are solely those of the authors and do not necessarily represent those of their affiliated organizations, or those of the publisher, the editors and the reviewers. Any product that may be evaluated in this article, or claim that may be made by its manufacturer, is not guaranteed or endorsed by the publisher.

## Footnotes

1
https://aussorgm.org.au/sorghum-qtl-atlas/

2
https://phytozome-next.jgi.doe.gov/info/Sbicolor_v3_1_1

3
https://data.jgi.doe.gov/refine-download/phytozome?q=Sorghum+bicolor&expanded=Phytozome-454

## Supplementary Material

Supplementary Figure 1 The KEEG enrichment for genes in blue module and turquois module. **(A)** The KEEG enrichment for genes in blue module. **(B)** The KEEG enrichment for genes in turquois module.
