# Supplementary figures and images for: Identification of Candidate Forage Yield Genes in Sorghum (Sorghum bicolor L.) Using Integrated Genome-Wide Association Studies and RNA-Seq
Source: Front Plant Sci. 2022 Jan 11;12:788433. doi: 10.3389/fpls.2021.788433 (PMC8787639; doi:10.3389/fpls.2021.788433)

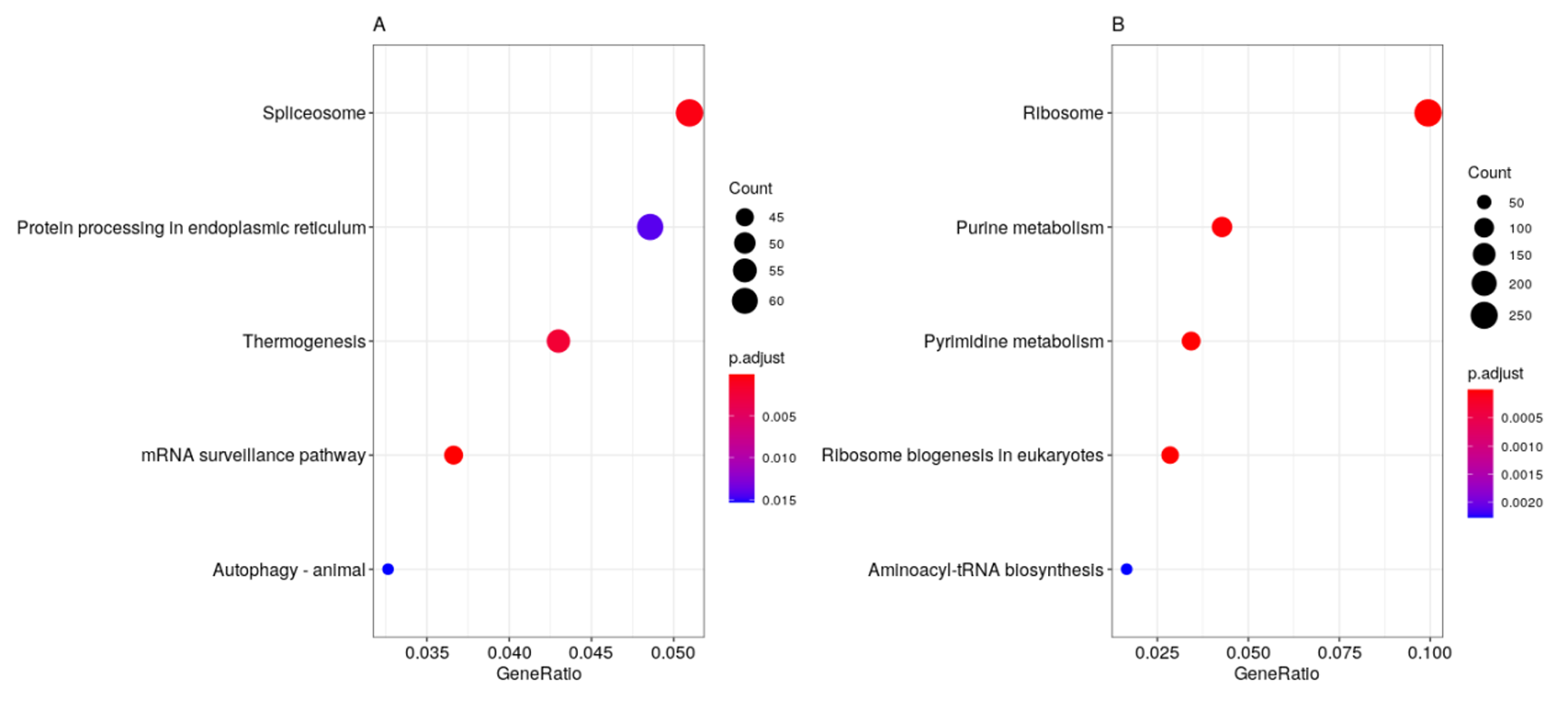

Supplement: Supplementary Figure 1 — The KEEG enrichment for genes in blue module and turquois module. (A) The KEEG enrichment for genes in blue module. (B) The KEEG enrichment for genes in turquois module. [file Image_1.tif]
