# Supplementary material for: Identification of Candidate Forage Yield Genes in Sorghum (Sorghum bicolor L.) Using Integrated Genome-Wide Association Studies and RNA-Seq
Source: Front Plant Sci. 2022 Jan 11;12:788433. doi: 10.3389/fpls.2021.788433 (PMC8787639; doi:10.3389/fpls.2021.788433)
Supplement: Supplementary file 2 [file Table_1.DOCX]

Table S1. The significant associated SNPs with PH, TN, SD and FW

| Trait | Marker | Chromosome | Position | P value | PVE | Environment |
| --- | --- | --- | --- | --- | --- | --- |
| PH | S1_2513147 | 1 | 2513147 | 2.04E-05 | 7.54% | PH-2015Fy |
|  | S1_4453796 |  | 4453796 | 3.39E-04 | 5.56% | PH -2016Tq |
|  | S1_10320621 |  | 10320621 | 8.51E-04 | 9.66% | PH -2017Tq |
|  | S1_41748958 |  | 41748958 | 8.42E-05 | 8.48% | PH -2016Tq |
|  | S1_46978339 |  | 46978339 | 2.20E-07 | 10.98% | PH-2015Fy |
|  | S1_46978339 |  | 46978339 | 8.71E-05 | 7.01% | PH -2016Fy |
|  | S1_63081303 |  | 63081303 | 5.93E-04 | 9.38% | PH -2017Tq |
|  | S2_6113781 | 2 | 6113781 | 4.18E-04 | 5.79% | PH -2017Tq |
|  | S2_6420786 |  | 6420786 | 2.32E-04 | 6.36% | PH -2016Tq |
|  | S2_6757652 |  | 6757652 | 8.89E-05 | 29.46% | PH-2015Fy |
|  | S2_65478636 |  | 65478636 | 5.38E-04 | 5.00% | PH -2016Tq |
|  | S2_66100213 |  | 66100213 | 4.01E-06 | 9.50% | PH -2016Fy |
|  | S2_69971881 |  | 69971881 | 7.86E-04 | 9.03% | PH -2017Tq |
|  | S2_75217621 |  | 75217621 | 2.79E-05 | 8.34% | PH-2015Fy |
|  | S3_4829992 | 3 | 4829992 | 1.96E-05 | 7.57% | PH-2015Fy |
|  | S3_4829992 |  | 4829992 | 6.20E-04 | 5.39% | PH -2016Fy |
|  | S3_10108607 |  | 10108607 | 2.10E-04 | 5.63% | PH -2016Tq |
|  | S3_23443068 |  | 23443068 | 5.14E-05 | 21.23% | PH -2017Tq |
|  | S3_39796457 |  | 39796457 | 3.41E-04 | 12.02% | PH -2016Fy |
|  | S3_48001728 |  | 48001728 | 1.31E-04 | 12.58% | PH -2017Tq |
|  | S3_51560390 |  | 51560390 | 3.40E-05 | 25.17% | PH -2016Tq |
|  | S3_66508420 |  | 66508420 | 1.68E-05 | 30.89% | PH-2015Fy |
|  | S3_73268694 |  | 73268694 | 1.68E-04 | 6.37% | PH -2016Fy |
|  | S4_19631727 | 4 | 19631727 | 7.38E-04 | 4.06% | PH -2017Tq |
|  | S4_24736629 |  | 24736629 | 9.08E-06 | 20.42% | PH -2016Tq |
|  | S4_25982379 |  | 25982379 | 7.75E-06 | 13.17% | PH-2015Fy |
|  | S4_38381169 |  | 38381169 | 1.29E-05 | 9.72% | PH -2016Tq |
|  | S4_43509891 |  | 43509891 | 8.73E-05 | 6.90% | PH -2016Fy |
|  | S4_43509891 |  | 43509891 | 4.36E-04 | 5.53% | PH -2017Tq |
|  | S4_57986321 |  | 57986321 | 2.31E-06 | 8.13% | PH-2015Fy |
|  | S5_2838247 | 5 | 2838247 | 4.52E-05 | 5.76% | PH-2015Fy |
|  | S5_8233029 |  | 8233029 | 4.75E-05 | 6.82% | PH -2016Fy |
|  | S5_12607515 |  | 12607515 | 2.13E-04 | 21.38% | PH -2016Tq |
|  | S5_41488997 |  | 41488997 | 1.40E-04 | 17.49% | PH -2016Tq |
|  | S5_42503720 |  | 42503720 | 9.67E-05 | 6.79% | PH-2015Fy |
|  | S5_56963340 |  | 56963340 | 8.62E-05 | 7.26% | PH -2016Fy |
|  | S5_61988242 |  | 61988242 | 7.34E-04 | 18.43% | PH -2017Tq |
|  | S6_2341875 | 6 | 2341875 | 4.72E-05 | 9.54% | PH-2015Fy |
|  | S6_6674642 |  | 6674642 | 8.99E-05 | 31.57% | PH -2016Tq |
|  | S6_41239459 |  | 41239459 | 7.09E-04 | 6.78% | PH -2017Tq |
|  | S6_46277261 |  | 46277261 | 1.50E-05 | 7.47% | PH -2016Tq |
|  | S6_48485667 |  | 48485667 | 1.40E-04 | 6.65% | PH -2016Fy |
|  | S6_52782956 |  | 52782956 | 4.19E-05 | 5.72% | PH-2015Fy |
|  | S7_5256904 | 7 | 5256904 | 1.44E-04 | 4.93% | PH-2015Fy |
|  | S7_5952111 |  | 5952111 | 1.00E-04 | 6.12% | PH -2016Tq |
|  | S7_6316621 |  | 6316621 | 4.59E-05 | 8.12% | PH -2017Tq |
|  | S7_6605897 |  | 6605897 | 2.67E-04 | 7.30% | PH -2016Fy |
|  | S7_23969481 |  | 23969481 | 6.66E-04 | 17.35% | PH -2016Fy |
|  | S7_54660566 |  | 54660566 | 1.35E-04 | 8.79% | PH -2016Tq |
|  | S7_62607809 |  | 62607809 | 2.90E-04 | 5.66% | PH-2015Fy |
|  | S7_63163798 |  | 63163798 | 8.93E-04 | 5.60% | PH -2016Fy |
|  | S8_869316 | 8 | 869316 | 2.69E-05 | 15.96% | PH -2016Tq |
|  | S8_943964 |  | 943964 | 8.16E-04 | 9.74% | PH -2016Fy |
|  | S8_977265 |  | 977265 | 2.55E-06 | 9.08% | PH-2015Fy |
|  | S8_39071155 |  | 39071155 | 5.73E-04 | 4.93% | PH -2016Tq |
|  | S8_52903433 |  | 52903433 | 3.34E-04 | 16.33% | PH -2016Fy |
|  | S8_53045404 |  | 53045404 | 6.20E-06 | 8.25% | PH-2015Fy |
|  | S8_53045404 |  | 53045404 | 5.31E-04 | 4.85% | PH -2016Tq |
|  | S9_2553818 | 9 | 2553818 | 6.60E-04 | 9.59% | PH -2016Fy |
|  | S9_4327702 |  | 4327702 | 1.45E-04 | 8.58% | PH-2015Fy |
|  | S9_22822760 |  | 22822760 | 8.87E-05 | 46.47% | PH -2016Tq |
|  | S9_42020204 |  | 42020204 | 8.74E-05 | 6.24% | PH -2016Tq |
|  | S9_47861278 |  | 47861278 | 3.06E-04 | 6.06% | PH -2016Fy |
|  | S9_56153113 |  | 56153113 | 6.67E-05 | 20.12% | PH -2016Tq |
|  | S9_58241455 |  | 58241455 | 4.46E-06 | 7.38% | PH-2015Fy |
|  | S10_2438010 | 10 | 2438010 | 2.51E-04 | 6.07% | PH -2016Tq |
|  | S10_2442170 |  | 2442170 | 6.71E-04 | 4.01% | PH-2015Fy |
|  | S10_11485786 |  | 11485786 | 8.94E-05 | 5.49% | PH -2017Tq |
|  | S10_11883775 |  | 11883775 | 2.38E-04 | 7.96% | PH -2016Fy |
|  | S10_52003709 |  | 52003709 | 8.76E-04 | 4.79% | PH -2017Tq |
|  | S10_56437482 |  | 56437482 | 4.83E-04 | 5.31% | PH-2015Fy |
|  | S10_56437490 |  | 56437490 | 9.38E-04 | 4.72% | PH -2016Tq |
|  | S10_58009096 |  | 58009096 | 8.38E-04 | 5.16% | PH -2016Fy |
|  | S10_58344932 |  | 58344932 | 4.11E-04 | 5.38% | PH-2015Fy |
| TN | S1_1623831 | 1 | 1623831 | 5.85E-06 | 10.12% | PH -2016Fy |
|  | S1_2464207 |  | 2464207 | 1.06E-04 | 8.57% | PH -2017Tq |
|  | S1_5360680 |  | 5360680 | 1.39E-05 | 16.67% | PH-2015Fy |
|  | S1_7260399 |  | 7260399 | 4.07E-08 | 32.40% | PH -2016Tq |
|  | S1_18306236 |  | 18306236 | 5.89E-06 | 15.45% | PH -2016Fy |
|  | S1_50047454 |  | 50047454 | 2.80E-04 | 36.23% | PH-2015Fy |
|  | S1_53399369 |  | 53399369 | 7.13E-05 | 31.00% | PH -2016Tq |
|  | S1_55184461 |  | 55184461 | 1.01E-04 | 34.21% | PH-2015Fy |
|  | S1_62800353 |  | 62800353 | 2.47E-05 | 8.11% | PH -2017Tq |
|  | S1_70264908 |  | 70264908 | 4.83E-06 | 10.31% | PH -2016Fy |
|  | S2_2220161 | 2 | 2220161 | 2.14E-06 | 14.97% | PH -2016Fy |
|  | S2_3359400 |  | 3359400 | 6.74E-05 | 7.21% | PH -2017Tq |
|  | S2_9657381 |  | 9657381 | 3.83E-05 | 7.60% | PH-2015Fy |
|  | S2_18195687 |  | 18195687 | 1.21E-05 | 56.33% | PH -2016Tq |
|  | S2_44793205 |  | 44793205 | 3.20E-04 | 16.55% | PH-2015Fy |
|  | S2_50326850 |  | 50326850 | 1.37E-05 | 9.20% | PH -2016Fy |
|  | S2_72379459 |  | 72379459 | 4.55E-05 | 10.61% | PH -2016Tq |
|  | S2_72379533 |  | 72379533 | 3.40E-05 | 7.68% | PH-2015Fy |
|  | S2_72469899 |  | 72469899 | 1.23E-06 | 11.67% | PH -2016Fy |
|  | S2_76517741 |  | 76517741 | 2.41E-05 | 8.05% | PH -2017Tq |
|  | S3_4624865 | 3 | 4624865 | 4.94E-07 | 16.71% | PH -2016Fy |
|  | S3_6239628 |  | 6239628 | 2.36E-06 | 35.73% | PH-2015Fy |
|  | S3_6239628 |  | 6239628 | 1.68E-05 | 23.39% | PH -2016Tq |
|  | S3_14891714 |  | 14891714 | 1.82E-06 | 17.39% | PH -2017Tq |
|  | S3_22562790 |  | 22562790 | 9.63E-05 | 34.13% | PH -2016Tq |
|  | S3_36343511 |  | 36343511 | 1.18E-05 | 24.23% | PH -2016Fy |
|  | S3_53982031 |  | 53982031 | 5.22E-07 | 29.10% | PH-2015Fy |
|  | S3_59245673 |  | 59245673 | 3.29E-07 | 12.71% | PH -2016Fy |
|  | S3_68669720 |  | 68669720 | 5.46E-08 | 16.59% | PH -2016Tq |
|  | S3_68669720 |  | 68669720 | 7.98E-07 | 13.84% | PH-2015Fy |
|  | S3_73198746 |  | 73198746 | 4.93E-05 | 7.38% | PH -2017Tq |
|  | S4_711535 | 4 | 711535 | 5.94E-06 | 9.40% | PH -2017Tq |
|  | S4_1261758 |  | 1261758 | 2.50E-07 | 11.44% | PH -2016Fy |
|  | S4_1261758 |  | 1261758 | 5.07E-06 | 9.59% | PH -2016Tq |
|  | S4_11018580 |  | 11018580 | 6.76E-05 | 9.00% | PH-2015Fy |
|  | S4_25369763 |  | 25369763 | 3.51E-05 | 13.89% | PH -2017Tq |
|  | S4_50570897 |  | 50570897 | 8.82E-06 | 57.07% | PH -2016Tq |
|  | S4_57019677 |  | 57019677 | 2.51E-04 | 8.58% | PH -2017Tq |
|  | S4_57961189 |  | 57961189 | 4.26E-06 | 25.23% | PH -2016Fy |
|  | S4_61455699 |  | 61455699 | 3.90E-05 | 7.39% | PH-2015Fy |
|  | S5_4584927 | 5 | 4584927 | 9.11E-05 | 23.29% | PH-2015Fy |
|  | S5_4584927 |  | 4584927 | 4.04E-04 | 14.02% | PH -2016Tq |
|  | S5_5692815 |  | 5692815 | 6.28E-05 | 9.84% | PH -2016Fy |
|  | S5_21076478 |  | 21076478 | 2.72E-04 | 6.45% | PH -2017Tq |
|  | S5_29355380 |  | 29355380 | 7.89E-05 | 49.68% | PH-2015Fy |
|  | S5_37351555 |  | 37351555 | 1.45E-05 | 12.32% | PH -2016Fy |
|  | S5_56024818 |  | 56024818 | 2.41E-04 | 7.12% | PH -2016Tq |
|  | S5_58269285 |  | 58269285 | 1.67E-06 | 11.25% | PH -2016Fy |
|  | S5_58642297 |  | 58642297 | 2.91E-06 | 9.79% | PH -2017Tq |
|  | S5_60343145 |  | 60343145 | 2.19E-04 | 41.16% | PH-2015Fy |
|  | S6_623843 | 6 | 623843 | 1.38E-05 | 9.87% | PH -2016Fy |
|  | S6_3164248 |  | 3164248 | 5.65E-04 | 5.89% | PH -2017Tq |
|  | S6_4353373 |  | 4353373 | 1.55E-04 | 10.49% | PH-2015Fy |
|  | S6_4368676 |  | 4368676 | 5.10E-05 | 17.64% | PH -2016Tq |
|  | S6_49710160 |  | 49710160 | 6.73E-06 | 9.06% | PH -2017Tq |
|  | S6_50419768 |  | 50419768 | 6.63E-06 | 9.28% | PH-2015Fy |
|  | S6_55885739 |  | 55885739 | 8.19E-07 | 13.79% | PH -2016Fy |
|  | S6_58395131 |  | 58395131 | 5.22E-05 | 7.55% | PH -2016Tq |
|  | S7_1764414 | 7 | 1764414 | 8.70E-07 | 23.16% | PH -2016Tq |
|  | S7_8099000 |  | 8099000 | 1.80E-06 | 14.14% | PH -2016Fy |
|  | S7_8852677 |  | 8852677 | 1.18E-04 | 10.94% | PH-2015Fy |
|  | S7_18366137 |  | 18366137 | 6.06E-04 | 16.21% | PH -2017Tq |
|  | S7_23084694 |  | 23084694 | 2.44E-04 | 8.49% | PH-2015Fy |
|  | S7_35676514 |  | 35676514 | 8.05E-06 | 9.78% | PH-2015Fy |
|  | S7_41434656 |  | 41434656 | 8.85E-04 | 26.45% | PH -2017Tq |
|  | S7_48988930 |  | 48988930 | 1.82E-05 | 18.59% | PH -2016Fy |
|  | S7_57527702 |  | 57527702 | 9.76E-06 | 8.73% | PH -2017Tq |
|  | S7_58337639 |  | 58337639 | 3.74E-05 | 7.42% | PH-2015Fy |
|  | S7_58337639 |  | 58337639 | 5.77E-05 | 6.85% | PH -2016Tq |
|  | S8_3557830 | 8 | 3557830 | 1.26E-04 | 6.94% | PH -2017Tq |
|  | S8_11808117 |  | 11808117 | 1.46E-05 | 12.60% | PH -2016Fy |
|  | S8_12150419 |  | 12150419 | 5.32E-04 | 12.79% | PH -2016Tq |
|  | S8_40603220 |  | 40603220 | 3.71E-06 | 22.21% | PH -2016Tq |
|  | S8_40603220 |  | 40603220 | 8.90E-06 | 18.21% | PH-2015Fy |
|  | S8_45028895 |  | 45028895 | 6.63E-07 | 18.40% | PH -2016Fy |
|  | S8_51327140 |  | 51327140 | 1.07E-04 | 6.93% | PH -2017Tq |
|  | S8_51327298 |  | 51327298 | 5.96E-06 | 9.23% | PH-2015Fy |
|  | S9_1599445 | 9 | 1599445 | 3.92E-06 | 15.70% | PH -2016Fy |
|  | S9_3261902 |  | 3261902 | 5.98E-05 | 7.46% | PH -2017Tq |
|  | S9_4170299 |  | 4170299 | 3.70E-05 | 8.38% | PH-2015Fy |
|  | S9_4170299 |  | 4170299 | 6.50E-05 | 7.53% | PH -2016Tq |
|  | S9_19908507 |  | 19908507 | 4.33E-06 | 32.63% | PH-2015Fy |
|  | S9_19908507 |  | 19908507 | 5.40E-05 | 25.77% | PH -2016Tq |
|  | S9_23157253 |  | 23157253 | 1.80E-05 | 10.65% | PH -2016Fy |
|  | S9_42349829 |  | 42349829 | 1.92E-05 | 17.92% | PH -2016Tq |
|  | S9_47678501 |  | 47678501 | 1.05E-05 | 10.43% | PH-2015Fy |
|  | S9_54463188 |  | 54463188 | 1.77E-05 | 8.87% | PH -2017Tq |
|  | S9_57704455 |  | 57704455 | 3.97E-05 | 8.42% | PH -2016Tq |
|  | S9_59391207 |  | 59391207 | 3.97E-08 | 14.77% | PH -2016Fy |
|  | S10_3436650 | 10 | 3436650 | 4.53E-04 | 7.04% | PH -2017Tq |
|  | S10_4454931 |  | 4454931 | 1.47E-07 | 20.82% | PH-2015Fy |
|  | S10_4454931 |  | 4454931 | 4.56E-06 | 14.13% | PH -2016Fy |
|  | S10_10594716 |  | 10594716 | 1.77E-05 | 41.35% | PH -2016Tq |
|  | S10_51545993 |  | 51545993 | 4.14E-05 | 7.36% | PH-2015Fy |
|  | S10_51545993 |  | 51545993 | 4.40E-05 | 7.64% | PH -2016Tq |
|  | S10_58248604 |  | 58248604 | 2.58E-05 | 8.08% | PH -2017Tq |
|  | S10_60100595 |  | 60100595 | 3.54E-08 | 15.01% | PH -2016Fy |
| SD | S1_884582 | 1 | 884582 | 1.43E-04 | 7.35% | PH -2016Tq |
|  | S1_4151656 |  | 4151656 | 2.37E-05 | 7.69% | PH-2015Fy |
|  | S1_5886330 |  | 5886330 | 4.60E-06 | 9.44% | PH -2016Fy |
|  | S1_14262128 |  | 14262128 | 1.45E-05 | 8.71% | PH -2017Tq |
|  | S1_20264990 |  | 20264990 | 2.97E-05 | 7.65% | PH-2015Fy |
|  | S1_60558038 |  | 60558038 | 2.09E-05 | 8.02% | PH -2016Fy |
|  | S1_68664964 |  | 68664964 | 7.44E-05 | 7.28% | PH-2015Fy |
|  | S1_69018661 |  | 69018661 | 1.34E-07 | 11.04% | PH -2017Tq |
|  | S1_72503695 |  | 72503695 | 8.35E-06 | 18.84% | PH -2016Tq |
|  | S2_3359388 | 2 | 3359388 | 3.33E-05 | 7.89% | PH -2016Fy |
|  | S2_6525373 |  | 6525373 | 2.90E-05 | 8.10% | PH-2015Fy |
|  | S2_16705281 |  | 16705281 | 6.30E-07 | 9.80% | PH -2017Tq |
|  | S2_23015150 |  | 23015150 | 3.48E-06 | 8.97% | PH -2016Tq |
|  | S2_41158462 |  | 41158462 | 1.42E-05 | 10.03% | PH -2016Tq |
|  | S2_58639879 |  | 58639879 | 2.54E-05 | 8.31% | PH -2017Tq |
|  | S2_64007374 |  | 64007374 | 3.83E-06 | 9.61% | PH -2016Fy |
|  | S2_77185213 |  | 77185213 | 2.93E-06 | 8.07% | PH-2015Fy |
|  | S3_4337503 | 3 | 4337503 | 7.62E-08 | 13.17% | PH -2017Tq |
|  | S3_4337503 |  | 4337503 | 4.65E-06 | 10.21% | PH -2016Tq |
|  | S3_11248735 |  | 11248735 | 6.41E-07 | 11.06% | PH-2015Fy |
|  | S3_18827146 |  | 18827146 | 7.27E-06 | 7.71% | PH -2016Fy |
|  | S3_66578380 |  | 66578380 | 6.51E-06 | 9.08% | PH -2016Fy |
|  | S3_66578380 |  | 66578380 | 1.03E-05 | 8.24% | PH-2015Fy |
|  | S3_69018585 |  | 69018585 | 1.38E-05 | 22.95% | PH -2017Tq |
|  | S3_69018585 |  | 69018585 | 1.95E-05 | 26.58% | PH -2016Tq |
|  | S4_612363 | 4 | 612363 | 4.40E-05 | 24.59% | PH -2016Tq |
|  | S4_686420 |  | 686420 | 3.49E-05 | 8.02% | PH -2017Tq |
|  | S4_2559427 |  | 2559427 | 4.31E-06 | 8.84% | PH-2015Fy |
|  | S4_6945517 |  | 6945517 | 1.15E-05 | 9.41% | PH -2016Fy |
|  | S4_34963394 |  | 34963394 | 4.39E-05 | 7.64% | PH -2016Fy |
|  | S4_53278153 |  | 53278153 | 5.99E-05 | 9.05% | PH -2016Fy |
|  | S4_60073437 |  | 60073437 | 5.68E-07 | 10.42% | PH -2017Tq |
|  | S4_65426066 |  | 65426066 | 2.21E-05 | 7.69% | PH-2015Fy |
|  | S4_65637434 |  | 65637434 | 1.29E-05 | 7.92% | PH -2016Tq |
|  | S5_2271046 | 5 | 2271046 | 6.58E-06 | 10.00% | PH -2017Tq |
|  | S5_3448516 |  | 3448516 | 6.63E-06 | 13.99% | PH -2016Fy |
|  | S5_3569277 |  | 3569277 | 5.93E-04 | 7.44% | PH -2016Tq |
|  | S5_15797888 |  | 15797888 | 6.67E-06 | 8.97% | PH-2015Fy |
|  | S5_44068288 |  | 44068288 | 3.71E-05 | 14.10% | PH -2016Tq |
|  | S5_46535707 |  | 46535707 | 1.55E-04 | 6.19% | PH-2015Fy |
|  | S5_55172163 |  | 55172163 | 3.53E-05 | 8.23% | PH -2017Tq |
|  | S5_55912627 |  | 55912627 | 5.02E-06 | 13.60% | PH -2016Fy |
|  | S6_384215 | 6 | 384215 | 5.50E-05 | 6.65% | PH -2016Tq |
|  | S6_1424215 |  | 1424215 | 1.85E-06 | 10.17% | PH -2016Fy |
|  | S6_1750164 |  | 1750164 | 5.06E-05 | 7.16% | PH-2015Fy |
|  | S6_5855896 |  | 5855896 | 3.44E-06 | 10.22% | PH -2017Tq |
|  | S6_49710265 |  | 49710265 | 1.14E-06 | 10.43% | PH -2016Fy |
|  | S6_49739130 |  | 49739130 | 8.79E-09 | 17.58% | PH -2017Tq |
|  | S6_51884630 |  | 51884630 | 6.71E-07 | 10.26% | PH-2015Fy |
|  | S6_52195082 |  | 52195082 | 3.53E-06 | 20.60% | PH -2016Tq |
|  | S7_157532 | 7 | 157532 | 3.04E-05 | 6.62% | PH-2015Fy |
|  | S7_541303 |  | 541303 | 7.15E-05 | 6.11% | PH -2017Tq |
|  | S7_3743390 |  | 3743390 | 3.96E-05 | 7.71% | PH -2016Fy |
|  | S7_5483499 |  | 5483499 | 3.95E-05 | 18.40% | PH -2016Tq |
|  | S7_55526214 |  | 55526214 | 5.16E-06 | 10.83% | PH -2016Tq |
|  | S7_55857920 |  | 55857920 | 2.22E-05 | 9.97% | PH-2015Fy |
|  | S7_55857920 |  | 55857920 | 2.23E-05 | 10.86% | PH -2016Fy |
|  | S7_62353657 |  | 62353657 | 5.25E-06 | 8.20% | PH -2017Tq |
|  | S8_977265 | 8 | 977265 | 1.28E-04 | 7.32% | PH -2016Tq |
|  | S8_5476974 |  | 5476974 | 7.82E-06 | 9.46% | PH -2017Tq |
|  | S8_11449350 |  | 11449350 | 1.77E-04 | 6.14% | PH-2015Fy |
|  | S8_11449359 |  | 11449359 | 1.56E-06 | 10.30% | PH -2016Fy |
|  | S8_52675870 |  | 52675870 | 1.00E-04 | 6.57% | PH-2015Fy |
|  | S8_53947324 |  | 53947324 | 1.14E-05 | 10.14% | PH -2017Tq |
|  | S8_55262749 |  | 55262749 | 5.50E-06 | 9.50% | PH -2016Fy |
|  | S8_55262749 |  | 55262749 | 8.73E-06 | 9.89% | PH -2016Tq |
|  | S9_1066326 | 9 | 1066326 | 4.19E-08 | 20.55% | PH -2017Tq |
|  | S9_1256103 |  | 1256103 | 1.41E-05 | 8.13% | PH -2016Fy |
|  | S9_4623251 |  | 4623251 | 8.31E-05 | 5.76% | PH-2015Fy |
|  | S9_13917693 |  | 13917693 | 2.54E-06 | 51.42% | PH -2016Tq |
|  | S9_49276623 |  | 49276623 | 1.80E-05 | 7.59% | PH -2017Tq |
|  | S9_51191811 |  | 51191811 | 7.30E-06 | 8.50% | PH-2015Fy |
|  | S9_52749388 |  | 52749388 | 4.23E-06 | 16.56% | PH -2016Fy |
|  | S9_55968631 |  | 55968631 | 6.13E-06 | 9.93% | PH -2016Tq |
|  | S10_6970450 | 10 | 6970450 | 2.49E-04 | 5.45% | PH -2016Tq |
|  | S10_9339186 |  | 9339186 | 4.27E-05 | 8.42% | PH -2016Fy |
|  | S10_12264109 |  | 12264109 | 4.05E-04 | 8.61% | PH-2015Fy |
|  | S10_17838081 |  | 17838081 | 3.04E-04 | 6.34% | PH -2017Tq |
|  | S10_37891701 |  | 37891701 | 1.83E-04 | 7.02% | PH -2016Tq |
|  | S10_51062530 |  | 51062530 | 2.30E-05 | 7.74% | PH-2015Fy |
|  | S10_54212511 |  | 54212511 | 3.35E-07 | 12.07% | PH -2017Tq |
|  | S10_54212511 |  | 54212511 | 1.67E-04 | 7.32% | PH -2016Tq |
|  | S10_60508113 |  | 60508113 | 7.00E-05 | 7.19% | PH -2016Fy |
| FW | S1_884582 | 1 | 884582 | 3.61E-05 | 8.08% | PH -2016Tq |
|  | S1_3555731 |  | 3555731 | 2.38E-04 | 24.57% | PH-2015Fy |
|  | S1_7355038 |  | 7355038 | 7.10E-05 | 7.45% | PH -2017Tq |
|  | S1_14903226 |  | 14903226 | 1.84E-11 | 18.52% | PH -2016Fy |
|  | S1_44861010 |  | 44861010 | 1.59E-07 | 11.45% | PH -2016Fy |
|  | S1_48631033 |  | 48631033 | 2.06E-09 | 21.04% | PH -2017Tq |
|  | S1_59587150 |  | 59587150 | 8.92E-05 | 7.80% | PH -2016Tq |
|  | S1_69138536 |  | 69138536 | 6.14E-08 | 11.83% | PH -2016Fy |
|  | S1_72602791 |  | 72602791 | 8.31E-06 | 7.86% | PH-2015Fy |
|  | S2_6023875 | 2 | 6023875 | 7.60E-05 | 6.18% | PH -2017Tq |
|  | S2_9525549 |  | 9525549 | 3.27E-04 | 5.54% | PH-2015Fy |
|  | S2_13416635 |  | 13416635 | 2.11E-11 | 20.60% | PH -2016Fy |
|  | S2_18281154 |  | 18281154 | 4.44E-05 | 8.49% | PH -2016Tq |
|  | S2_50022890 |  | 50022890 | 1.02E-08 | 40.11% | PH -2016Fy |
|  | S2_67383083 |  | 67383083 | 1.22E-06 | 10.78% | PH -2017Tq |
|  | S2_69088304 |  | 69088304 | 1.87E-04 | 6.93% | PH -2016Tq |
|  | S2_76681623 |  | 76681623 | 3.19E-04 | 8.45% | PH-2015Fy |
|  | S2_76938506 |  | 76938506 | 5.03E-07 | 10.27% | PH -2016Fy |
|  | S3_4644100 | 3 | 4644100 | 3.10E-05 | 7.46% | PH -2016Fy |
|  | S3_7584612 |  | 7584612 | 4.37E-04 | 4.99% | PH-2015Fy |
|  | S3_8395829 |  | 8395829 | 4.22E-04 | 33.04% | PH -2017Tq |
|  | S3_14441873 |  | 14441873 | 2.96E-04 | 12.71% | PH -2016Tq |
|  | S3_42150424 |  | 42150424 | 4.23E-05 | 10.08% | PH -2016Fy |
|  | S3_59587494 |  | 59587494 | 4.65E-04 | 6.42% | PH -2017Tq |
|  | S3_65798168 |  | 65798168 | 3.25E-04 | 6.40% | PH -2016Tq |
|  | S3_68868382 |  | 68868382 | 9.58E-08 | 12.99% | PH -2017Tq |
|  | S3_69297702 |  | 69297702 | 7.05E-08 | 22.25% | PH -2016Fy |
|  | S3_72703126 |  | 72703126 | 2.60E-04 | 8.08% | PH-2015Fy |
|  | S4_766971 | 4 | 766971 | 4.76E-07 | 44.86% | PH -2016Fy |
|  | S4_4121226 |  | 4121226 | 1.11E-04 | 8.25% | PH-2015Fy |
|  | S4_7823153 |  | 7823153 | 3.66E-07 | 10.39% | PH -2017Tq |
|  | S4_20870345 |  | 20870345 | 5.78E-04 | 7.26% | PH -2016Tq |
|  | S4_42603035 |  | 42603035 | 7.24E-10 | 45.68% | PH -2016Fy |
|  | S4_53242453 |  | 53242453 | 6.31E-05 | 6.25% | PH-2015Fy |
|  | S4_61397057 |  | 61397057 | 1.63E-04 | 6.97% | PH -2017Tq |
|  | S4_64875293 |  | 64875293 | 2.82E-04 | 18.44% | PH -2016Tq |
|  | S5_6111609 | 5 | 6111609 | 8.64E-05 | 7.78% | PH -2017Tq |
|  | S5_11201223 |  | 11201223 | 1.32E-04 | 6.02% | PH-2015Fy |
|  | S5_13334304 |  | 13334304 | 7.91E-06 | 8.43% | PH -2016Fy |
|  | S5_17061162 |  | 17061162 | 3.50E-04 | 7.81% | PH -2016Tq |
|  | S5_22469378 |  | 22469378 | 5.94E-04 | 14.55% | PH -2017Tq |
|  | S5_44596106 |  | 44596106 | 5.45E-04 | 19.45% | PH-2015Fy |
|  | S5_49656397 |  | 49656397 | 1.25E-11 | 18.57% | PH -2016Fy |
|  | S5_55172133 |  | 55172133 | 4.60E-05 | 7.96% | PH -2016Tq |
|  | S5_59917760 |  | 59917760 | 6.38E-07 | 12.81% | PH -2017Tq |
|  | S6_3556255 | 6 | 3556255 | 1.33E-06 | 25.31% | PH -2016Fy |
|  | S6_10554339 |  | 10554339 | 5.67E-06 | 18.78% | PH -2017Tq |
|  | S6_43206544 |  | 43206544 | 2.87E-04 | 6.56% | PH-2015Fy |
|  | S6_47193571 |  | 47193571 | 7.90E-12 | 20.31% | PH -2016Fy |
|  | S6_50567162 |  | 50567162 | 2.78E-04 | 10.62% | PH -2017Tq |
|  | S6_50622038 |  | 50622038 | 3.70E-04 | 5.99% | PH-2015Fy |
|  | S6_58247716 |  | 58247716 | 1.51E-04 | 6.68% | PH -2016Tq |
|  | S7_3730288 | 7 | 3730288 | 4.72E-06 | 8.62% | PH-2015Fy |
|  | S7_5483499 |  | 5483499 | 1.49E-04 | 14.28% | PH -2016Tq |
|  | S7_7789099 |  | 7789099 | 2.41E-05 | 34.70% | PH -2017Tq |
|  | S7_16534599 |  | 16534599 | 9.88E-10 | 39.06% | PH -2016Fy |
|  | S7_53546648 |  | 53546648 | 1.70E-04 | 5.82% | PH-2015Fy |
|  | S7_61868653 |  | 61868653 | 2.85E-06 | 11.96% | PH -2016Fy |
|  | S7_61937678 |  | 61937678 | 2.76E-08 | 14.03% | PH -2017Tq |
|  | S7_62124405 |  | 62124405 | 3.83E-04 | 5.96% | PH -2016Tq |
|  | S8_3176677 | 8 | 3176677 | 6.62E-05 | 8.01% | PH -2016Tq |
|  | S8_7437772 |  | 7437772 | 5.55E-06 | 21.64% | PH -2017Tq |
|  | S8_11923031 |  | 11923031 | 1.73E-04 | 5.79% | PH-2015Fy |
|  | S8_11923031 |  | 11923031 | 5.42E-04 | 5.15% | PH -2016Fy |
|  | S8_42203421 |  | 42203421 | 3.66E-04 | 9.95% | PH -2016Fy |
|  | S8_49743378 |  | 49743378 | 1.62E-04 | 11.44% | PH-2015Fy |
|  | S8_51943027 |  | 51943027 | 7.52E-06 | 9.40% | PH -2016Tq |
|  | S8_53682969 |  | 53682969 | 1.72E-04 | 5.45% | PH -2017Tq |
|  | S9_1118812 | 9 | 1118812 | 1.95E-11 | 21.59% | PH -2016Fy |
|  | S9_1194513 |  | 1194513 | 4.33E-04 | 10.90% | PH -2017Tq |
|  | S9_4891135 |  | 4891135 | 8.63E-06 | 9.16% | PH -2016Tq |
|  | S9_6396425 |  | 6396425 | 6.55E-04 | 4.69% | PH-2015Fy |
|  | S9_44660188 |  | 44660188 | 1.48E-04 | 6.47% | PH -2016Fy |
|  | S9_47124339 |  | 47124339 | 1.83E-04 | 6.52% | PH -2016Tq |
|  | S9_50573691 |  | 50573691 | 1.35E-04 | 6.94% | PH -2017Tq |
|  | S9_56641111 |  | 56641111 | 1.90E-04 | 14.60% | PH-2015Fy |
|  | S10_130955 | 10 | 130955 | 9.08E-05 | 6.00% | PH-2015Fy |
|  | S10_5105204 |  | 5105204 | 1.38E-04 | 6.42% | PH -2016Fy |
|  | S10_17838081 |  | 17838081 | 1.28E-05 | 8.72% | PH -2016Tq |
|  | S10_20516647 |  | 20516647 | 1.10E-04 | 10.07% | PH-2015Fy |
|  | S10_45845601 |  | 45845601 | 2.08E-05 | 7.28% | PH-2015Fy |
|  | S10_49279388 |  | 49279388 | 8.81E-04 | 7.19% | PH -2017Tq |
|  | S10_55671820 |  | 55671820 | 6.24E-04 | 10.80% | PH -2016Fy |
|  | S10_55747741 |  | 55747741 | 2.62E-04 | 6.47% | PH -2016Tq |
