# Supplementary material for: Identification of Candidate Forage Yield Genes in Sorghum (Sorghum bicolor L.) Using Integrated Genome-Wide Association Studies and RNA-Seq
Source: Front Plant Sci. 2022 Jan 11;12:788433. doi: 10.3389/fpls.2021.788433 (PMC8787639; doi:10.3389/fpls.2021.788433)
Supplement: Supplementary file 3 [file Table_2.DOCX]

Table S2. The annotated candidate genes for PH, TN, SD

| Trait | Chromosome | Interval_start | Interval_end | gene_start | gene_end | gene number | gene name |
| --- | --- | --- | --- | --- | --- | --- | --- |
| PH | Chr03 | 4804992 | 4854992 | 4811056 | 4811927 | Sobic.003G053001 | NA |
|  |  |  |  | 4826319 | 4826971 | Sobic.003G053100 | NA |
|  |  |  |  | 4829807 | 4831415 | Sobic.003G053200 | MYB family transcription factor, |
|  |  |  |  | 4847599 | 4848761 | Sobic.003G053300 | MYB family transcription factor, |
|  |  |  |  | 4852586 | 4854806 | Sobic.003G053400 | hydrolase, alpha/beta fold family protein, |
|  |  | 6214628 | 6264628 | 6219425 | 6220717 | Sobic.003G073166 | expressed protein |
|  | Chr04 | 43484891 | 43534891 | 43520796 | 43529651 | Sobic.004G143900 | indole-3-glycerol phosphate synthase, chloroplast precursor, |
|  | Chr08 | 53020404 | 53070404 | 11930915 | 11935547 | Sobic.008G077600 | diacylglycerol kinase 1, |
|  |  |  |  | 53044929 | 53050870 | Sobic.008G118100 | kelch repeat protein, |
|  |  |  |  | 53059491 | 53066424 | Sobic.008G118200 | vesicle tethering family protein, |
|  |  |  |  | 55243892 | 55247353 | Sobic.008G127550 | pentatricopeptide repeat domain containing protein, |
|  |  |  |  | 55255389 | 55256948 | Sobic.008G127600 | MBTB5 - Bric-a-Brac, Tramtrack, Broad Complex BTB domain with Meprin and TRAF Homology MATH domain, expressed |
|  |  |  |  | 55273895 | 55275309 | Sobic.008G127700 | MBTB2 - Bric-a-Brac, Tramtrack, Broad Complex BTB domain with Meprin and TRAF Homology MATH domain, expressed |
|  |  |  |  | 55282479 | 55284855 | Sobic.008G127800 | MBTB64 - Bric-a-Brac, Tramtrack, Broad Complex BTB domain with Meprin and TRAF Homology MATH domain, expressed |
| TN | Chr03 | 6214628 | 6264628 | 6232381 | 6233550 | Sobic.003G073200 | hypothetical protein |
|  |  |  |  | 6241321 | 6244297 | Sobic.003G073300 | expressed protein |
|  |  |  |  | 6247862 | 6249076 | Sobic.003G073380 | hypothetical protein |
|  |  |  |  | 6253919 | 6254269 | Sobic.003G073460 | NA |
|  |  |  |  | 6262232 | 6262846 | Sobic.003G073540 | NA |
|  |  | 68644720 | 68694720 | 68677034 | 68680283 | Sobic.003G370700 | ZOS1-18 - C2H2 zinc finger protein, expressed |
|  |  |  |  | 68681474 | 68684666 | Sobic.003G370800 | aminotransferase, classes I and II, domain containing protein, expressed |
|  |  |  |  | 68684974 | 68687499 | Sobic.003G370900 | expressed protein |
|  | Chr04 | 1236758 | 1286758 | 1237474 | 1239623 | Sobic.004G015400 | AMP-binding enzyme, |
|  |  |  |  | 1241456 | 1246803 | Sobic.004G015500 | receptor kinase, |
|  |  |  |  | 1247474 | 1248927 | Sobic.004G015600 | NA |
|  |  |  |  | 1247861 | 1248536 | Sobic.004G015550 | NA |
|  |  |  |  | 1251084 | 1258462 | Sobic.004G015700 | serine/threonine-protein kinase receptor precursor, |
|  |  |  |  | 1260107 | 1265494 | Sobic.004G015800 | zinc carboxypeptidase family protein, |
|  |  |  |  | 1267957 | 1273130 | Sobic.004G015900 | expressed protein |
|  |  |  |  | 1275710 | 1281871 | Sobic.004G016000 | spermidine synthase, |
|  |  |  |  | 1283356 | 1287120 | Sobic.004G016100 | expressed protein |
|  | Chr05 | 4559927 | 4609927 | 4560412 | 4563928 | Sobic.005G047800 | NBS-LRR disease resistance protein, |
|  |  |  |  | 4589741 | 4592626 | Sobic.005G047900 | expressed protein |
|  |  |  |  | 4594880 | 4598680 | Sobic.005G048100 | NA |
|  |  |  |  | 4596972 | 4597556 | Sobic.005G048000 | WD domain, G-beta repeat domain containing protein, expressed |
|  |  |  |  | 4608163 | 4614745 | Sobic.005G048200 | expressed protein |
|  | Chr07 | 58312639 | 58362639 | 55875048 | 55876007 | Sobic.007G134140 | NA |
|  |  |  |  | 58313210 | 58314008 | Sobic.007G151200 | Cupin domain containing protein, expressed |
|  |  |  |  | 58317061 | 58318749 | Sobic.007G151250 | expressed protein |
|  |  |  |  | 58321851 | 58322842 | Sobic.007G151300 | retrotransposon protein, putative, unclassified, expressed |
|  |  |  |  | 58341777 | 58345411 | Sobic.007G151400 | cytokinin dehydrogenase precursor, |
|  | Chr09 | 4145299 | 4195299 | 4145185 | 4146327 | Sobic.009G043700 | glutathione S-transferase, |
|  |  |  |  | 4148531 | 4151725 | Sobic.009G043800 | dihydroflavonol-4-reductase, |
|  | Chr10 | 4429931 | 4479931 | 4429886 | 4430970 | Sobic.010G056800 | NA |
|  |  |  |  | 4441538 | 4442682 | Sobic.010G056900 | NA |
|  |  |  |  | 4450365 | 4451102 | Sobic.010G057000 | OsCML30 - Calmodulin-related calcium sensor protein, expressed |
|  |  |  |  | 4456582 | 4457405 | Sobic.010G057100 | expressed protein |
|  |  |  |  | 4458519 | 4459860 | Sobic.010G057200 | NA |
|  |  |  |  | 4465358 | 4466036 | Sobic.010G057250 | NA |
|  |  |  |  | 4474788 | 4478602 | Sobic.010G057300 | uncharacterized glycosyltransferase, |
|  |  | 51520993 | 51570993 | 51531281 | 51534517 | Sobic.010G177400 | dehydrogenase/reductase SDR family member 12, |
|  |  |  |  | 51538507 | 51540213 | Sobic.010G177500 | syntaxin, |
|  |  |  |  | 51547535 | 51549353 | Sobic.010G177600 | glucan endo-1,3-beta-glucosidase precursor, |
|  |  |  |  | 51567424 | 51568369 | Sobic.010G177800 | RALFL28 - Rapid ALkalinization Factor RALF family protein precursor, expressed |
| SD | Chr03 | 4312503 | 4362503 | 4310958 | 4314684 | Sobic.003G047200 | expressed protein |
|  |  |  |  | 4314744 | 4318941 | Sobic.003G047300 | phototropic-responsive NPH3 family protein, |
|  |  |  |  | 4321655 | 4323859 | Sobic.003G047400 | pentatricopeptide, |
|  |  |  |  | 4324003 | 4331419 | Sobic.003G047450 | NA |
|  |  |  |  | 4324382 | 4326989 | Sobic.003G047500 | flavonol-3-O-glycoside-7-O-glucosyltransferase 1, |
|  |  |  |  | 4331724 | 4334014 | Sobic.003G047600 | flavonol-3-O-glycoside-7-O-glucosyltransferase 1, |
|  |  |  |  | 4344453 | 4346355 | Sobic.003G047700 | cytokinin-O-glucosyltransferase 3, |
|  |  |  |  | 4347677 | 4350277 | Sobic.003G047800 | cytokinin-O-glucosyltransferase 3, |
|  |  | 66553380 | 66603380 | 66565838 | 66579954 | Sobic.003G344200 | leaf senescence related protein, |
|  |  |  |  | 66568770 | 66569614 | Sobic.003G344300 | NA |
|  |  |  |  | 66578571 | 66579143 | Sobic.003G344400 | zinc finger, C3HC4 type domain containing protein, expressed |
|  |  |  |  | 66583460 | 66584226 | Sobic.003G344501 | helix-loop-helix DNA-binding domain containing protein, expressed |
|  |  |  |  | 66595635 | 66598918 | Sobic.003G344600 | BAG domain containing protein, expressed |
|  |  |  |  | 66599390 | 66602632 | Sobic.003G344700 | ammonium transporter protein, |
|  |  |  |  | 66602544 | 66606974 | Sobic.003G344800 | phosphatidate cytidylyltransferase, |
|  |  |  |  | 68647071 | 68657566 | Sobic.003G370500 | hydrolase, alpha/beta fold family domain containing protein, expressed |
|  |  |  |  | 68665573 | 68672699 | Sobic.003G370600 | hydrolase, alpha/beta fold family domain containing protein, expressed |
|  |  | 68993585 | 69043585 | 68988981 | 68994567 | Sobic.003G375000 | RNA recognition motif containing protein, |
|  |  |  |  | 68995396 | 68998157 | Sobic.003G375100 | snRK1-interacting protein 1, |
|  |  |  |  | 68999883 | 69007574 | Sobic.003G375200 | mitotic checkpoint family protein, |
|  |  |  |  | 69008000 | 69010834 | Sobic.003G375300 | galactosyltransferase, |
|  |  |  |  | 69012217 | 69013242 | Sobic.003G375400 | NA |
|  |  |  |  | 69015375 | 69018970 | Sobic.003G375500 | PHD-finger domain containing protein, |
|  |  |  |  | 69022007 | 69022443 | Sobic.003G375600 | NA |
|  |  |  |  | 69028768 | 69031390 | Sobic.003G375700 | expressed protein |
|  |  |  |  | 69041682 | 69047570 | Sobic.003G375800 | receptor-like protein kinase HAIKU2 precursor, |
|  | Chr10 | 54187511 | 54237511 | 54187083 | 54187803 | Sobic.010G198700 | expressed protein |
|  |  |  | 54237511 | 54191191 | 54194198 | Sobic.010G198800 | expressed protein |
|  |  |  | 54237511 | 54204520 | 54206418 | Sobic.010G198900 | phytosulfokines 1 precursor, |
|  |  |  | 54237511 | 54227450 | 54228046 | Sobic.010G199000 | bZIP transcription factor domain containing protein, expressed |
|  |  |  | 54237511 | 54231210 | 54232441 | Sobic.010G199100 | zinc finger, C3HC4 type domain containing protein, expressed |
|  |  |  | 54237511 | 54233583 | 54235989 | Sobic.010G199200 | amino acid transporter, |
