# Supplementary material for: Identification of Candidate Forage Yield Genes in Sorghum (Sorghum bicolor L.) Using Integrated Genome-Wide Association Studies and RNA-Seq
Source: Front Plant Sci. 2022 Jan 11;12:788433. doi: 10.3389/fpls.2021.788433 (PMC8787639; doi:10.3389/fpls.2021.788433)
Supplement: Supplementary file 4 [file Table_3.DOCX]

Table S3. The overlapped QTNs with previous researches

| Trait | Marker | Chromosome | Position | p1/p2 | PVE1/PVE2 | QTL Id | LG:Start-End | Publication |
| --- | --- | --- | --- | --- | --- | --- | --- | --- |
| PH | S4_43509891 | 4 | 43,509,891 | 8.73E-05/4.36E-04 | 6.89%/5.53% | QHGHT4.2 | 4:2,655,500-4,670,409 | Madhusudhana and Patil, 2012 |
|  | S8_53045404 | 8 | 53,045,404 | 6.20E-06/5.31E-04 | 8.25%/4.85% | QHGHT8.1 | 8:5,070,472-57,519,538 | Mocoeur et al., 2015 |
|  |  |  |  |  |  | QHGHT8.3 | 8:53,884,235-56,649,242 | Phuong et al., 2013 |
| TN | S3_6239628 | 3 | 6,239,628 | 2.36E-06/1.68E-05 | 20.82%/14.13% | QTNUM3.4 | 3:5,993,979-7,936,069 | Kong et al., 2014 |
|  | S3_68669720 | 3 | 68,669,720 | 7.98E-07/5.46E-08 | 7.36%/7.64% | QTNUM3.12 | 3:62,348,624-69,835,316 | Mocoeur et al., 2015 |
|  |  |  |  |  |  | QTNUM3.10 | 3:64,160,917-68,201,560 | Shiringani et al., 2010 |
|  |  |  |  |  |  | QTNUM3.18 | 3:66,166,112-71,465,967 | Liu et al., 2019 |
|  | S7_58337639 | 7 | 58,337,639 | 3.74E-05/5.77E-05 | 9.59%/11.44% | QTNUM7.4 | 7:7,540,868-58,184,111 | Kong et al., 2014 |
|  |  |  |  |  |  | QTNUM7.5 | 7:9,128,540-58,270,104 | Paterson et al., 1995 |
|  | S9_19908507 | 9 | 19,908,507 | 4.33E-06/5.40E-05 | 7.42%/6.85% | QTNUM9.4 | 9:10,801,023-53,893,383 | Kong et al., 2014 |
|  | S10_4454931 | 10 | 4,454,931 | 1.47E-07/4.56E-06 | 32.63%/25.77% | QTNUM10.8 | 10:1,589,635-7,643,988 | Mocoeur et al., 2015 |
|  | S10_51545993 | 10 | 51,545,993 | 4.14E-05/4.40E-05 | 8.38%/7.53% | QTNUM10.9 | 10:51,108,171-51,679,429 | Zhao et al., 2016 |
| SD | S3_4337503 | 3 | 4,337,503 | 4.65E-06/7.62E-08 | 10.21%/13.17% | QSTDM3.2 | 3:0-5,415,510 | Shehzad and Okuno, 2015 |
|  | S3_66578380 | 3 | 66,578,380 | 1.03E-05/6.51E-06 | 8.24%/9.08% | QSTDM3.4 | 3:63,660,172-68,225,538 | Shiringani et al., 2010 |
|  | S3_69018585 | 3 | 69,018,585 | 1.95E-05/1.38E-05 | 26.58%/22.95% | QSTDM3.5 | 3:68,127,393-69,146,412 | Phuong et al., 2013 |
|  | S8_55262749 | 8 | 55,262,749 | 8.73E-06/5.50E-06 | 9.89%/9.50% | QSTDM8.1 | 8:55,702,264-57,553,924 | Shiringani et al., 2010 |

Reference:

Kong, W., Guo, H., Goff, V. H., Lee, T.-H., Kim, C., and Paterson, A. H. (2014). Genetic analysis of vegetative branching in sorghum. *Theor Appl Genet* 127, 2387–2403. doi:10.1007/s00122-014-2384-x.

Liu, H., Liu, H., Zhou, L., and Lin, Z. (2019). Genetic Architecture of domestication- and improvement-related traits using a population derived from Sorghum virgatum and Sorghum bicolor. *Plant Sci* 283, 135–146. doi:10.1016/j.plantsci.2019.02.013.

Madhusudhana, R., and Patil, J. (2012). A major QTL for plant height is linked with bloom locus in sorghum [Sorghum bicolor (L.) Moench]. *Euphytica* 191, 259–268.

Mocoeur, A., Zhang, Y.-M., Liu, Z.-Q., Shen, X., Zhang, L.-M., Rasmussen, S. K., et al. (2015). Stability and genetic control of morphological, biomass and biofuel traits under temperate maritime and continental conditions in sweet sorghum (Sorghum bicolour). *Theor Appl Genet* 128, 1685–1701. doi:10.1007/s00122-015-2538-5.

Paterson, A. H., Schertz, K. F., Lin, Y. R., Liu, S. C., and Chang, Y. L. (1995). The weediness of wild plants: molecular analysis of genes influencing dispersal and persistence of johnsongrass, Sorghum halepense (L.) Pers. *Proc Natl Acad Sci U S A* 92, 6127–6131. doi:10.1073/pnas.92.13.6127.

Phuong, N., Stützel, H., and Uptmoor, R. (2013). Quantitative trait loci associated to agronomic traits and yield components in a &lt;i&gt;Sorghum bicolor&lt;/i&gt; L. Moench RIL population cultivated under pre-flowering drought and well-watered conditions. *AS* 04, 781–791. doi:10.4236/as.2013.412107.

Shehzad, T., and Okuno, K. (2015). QTL mapping for yield and yield-contributing traits in sorghum (Sorghum bicolor (L.) Moench) with genome-based SSR markers. *Euphytica* 203, 17–31. doi:10.1007/s10681-014-1243-9.

Shiringani, A. L., Frisch, M., and Friedt, W. (2010). Genetic mapping of QTLs for sugar-related traits in a RIL population of Sorghum bicolor L. Moench. *Theor Appl Genet* 121, 323–336. doi:10.1007/s00122-010-1312-y.

Zhao, J., Mantilla Perez, M. B., Hu, J., and Salas Fernandez, M. G. (2016). Genome-Wide Association Study for Nine Plant Architecture Traits in Sorghum. *Plant Genome* 9. doi:10.3835/plantgenome2015.06.0044.
