# Supplementary material for: Identification of Candidate Forage Yield Genes in Sorghum (Sorghum bicolor L.) Using Integrated Genome-Wide Association Studies and RNA-Seq
Source: Front Plant Sci. 2022 Jan 11;12:788433. doi: 10.3389/fpls.2021.788433 (PMC8787639; doi:10.3389/fpls.2021.788433)
Supplement: Supplementary file 6 [file Table_5.DOCX]

Table S5 The shared DGEs classified to gene modules by WGCNA

| **Gene_id** | **Module** | **Gene name** | **stage1_stage2** | **stage1_stage2** | **stage2_stage3** | **stage2_stage3** | **stage3_stage4** | **stage3_stage4** |
| --- | --- | --- | --- | --- | --- | --- | --- | --- |
|  |  |  | **log2(FC)** | **direction** | **log2(FC)** | **direction** | **log2(FC)** | **direction** |
| Sobic.001G259900 | turquoise | jasmonate-zim-domain protein 1 | -6.35 | down | 6.88 | up | -8.14 | down |
| Sobic.008G036000 | turquoise | cytochrome P450, family 94, subfamily C,  polypeptide 1 | -6.81 | down | 7.51 | up | -6.88 | down |
| Sobic.004G191400 | turquoise | NA | -6.93 | down | 5.59 | up | -6.41 | down |
| Sobic.002G345200 | turquoise | Chaperonin-like RbcX protein | -2.27 | down | -3.58 | down | -5.87 | down |
| Sobic.003G088400 | turquoise | NA | -6.04 | down | 4.29 | up | -5.37 | down |
| Sobic.001G259600 | turquoise | jasmonate-zim-domain protein 11 | -3.74 | down | 4.71 | up | -5.04 | down |
| Sobic.004G280900 | turquoise | brassinosteroid-responsive RING-H2 | -4.67 | down | 4.18 | up | -4.92 | down |
| Sobic.001G259700 | turquoise | jasmonate-zim-domain protein 11 | -6.12 | down | 6.19 | up | -4.77 | down |
| Sobic.008G075400 | turquoise | NA | -6.72 | down | 2.98 | up | -4.54 | down |
| Sobic.001G519700 | green | Nucleotide-diphospho-sugar transferase  family protein | 3.66 | up | -4.96 | down | 4.33 | up |
| Sobic.005G102300 | turquoise | Disease resistance-responsive  (dirigent-like protein) family protein | -8.53 | down | 5.58 | up | -4.15 | down |
| Sobic.003G330100 | turquoise | Ribonuclease III family protein | -2.02 | down | -1.70 | down | -3.56 | down |
| Sobic.006G005600 | black | heat shock protein 90.1 | 3.68 | up | 4.83 | up | 3.51 | up |
| Sobic.001G425500 | black | HSP20-like chaperones superfamily protein | 6.77 | up | 2.76 | up | 3.46 | up |
| Sobic.009G119200 | yellow | Glycosyl hydrolase superfamily protein | 3.17 | up | -4.69 | down | 3.46 | up |
| Sobic.006G163401 | white | Protein kinase superfamily protein | -2.26 | down | 4.11 | up | -3.39 | down |
| Sobic.002G093500 | turquoise | Peptidase M20/M25/M40 family protein | -1.93 | down | -1.32 | down | -3.35 | down |
| Sobic.001G357100 | turquoise | zinc finger protein 3 | -5.73 | down | 3.99 | up | -3.22 | down |
| Sobic.001G482700 | turquoise | jasmonate-zim-domain protein 1 | -2.80 | down | 3.32 | up | -3.18 | down |
| Sobic.009G075100 | turquoise | Polyketide cyclase/dehydrase and lipid transport superfamily protein | -2.77 | down | -1.18 | down | -3.08 | down |
| Sobic.005G109000 | turquoise | cytochrome P450, family 94,  subfamily B, polypeptide 3 | -4.87 | down | 3.39 | up | -3.03 | down |
| Sobic.007G047450 | blue | NA | 7.64 | up | 2.60 | up | 3.01 | up |
| Sobic.009G254600 | cyan | alpha/beta-Hydrolases superfamily protein | -2.84 | down | 3.41 | up | -3.00 | down |
| Sobic.003G248901 | turquoise | NA | -7.73 | down | 3.47 | up | -2.98 | down |
| Sobic.001G456400 | midnightblue | ABI five binding protein 3 | -1.82 | down | 2.56 | up | -2.98 | down |
| Sobic.005G080200 | turquoise | Calcineurin-like metallo-phosphoesterase  superfamily protein | -5.50 | down | 3.31 | up | -2.97 | down |
| Sobic.003G221700 | turquoise | Mog1/PsbP/DUF1795-like photosystem II  reaction center PsbP family protein | -1.10 | down | -1.56 | down | -2.96 | down |
| Sobic.003G151100 | green | NA | 4.24 | up | -4.92 | down | 2.93 | up |
| Sobic.007G227600 | turquoise | RNA-binding (RRM/RBD/RNP motifs)  family protein | -2.52 | down | -1.97 | down | -2.92 | down |
| Sobic.009G031100 | purple | cyclopropyl isomerase | -1.18 | down | -1.49 | down | 2.78 | up |
| Sobic.001G095700 | turquoise | basic helix-loop-helix (bHLH) DNA-binding  superfamily protein | -3.35 | down | 2.76 | up | -2.74 | down |
| Sobic.001G482600 | midnightblue | jasmonate-zim-domain protein 1 | -2.76 | down | 3.59 | up | -2.74 | down |
| Sobic.002G038300 | turquoise | chloroplast RNA-binding protein 33 | -1.20 | down | -1.83 | down | -2.70 | down |
| Sobic.004G064400 | turquoise | Class I glutamine amidotransferase-like  superfamily protein | -2.33 | down | 2.95 | up | -2.67 | down |
| Sobic.005G138600 | turquoise | DEAD/DEAH box helicase, putative | -1.45 | down | -2.70 | down | -2.62 | down |
| Sobic.001G323701 | yellow | CCCH-type zinc fingerfamily protein with  RNA-binding domain | 4.20 | up | -3.63 | down | 2.51 | up |
| Sobic.005G042700 | yellow | ovate family protein 13 | 1.64 | up | -2.32 | down | -2.47 | down |
| Sobic.006G056400 | turquoise | jasmonate-zim-domain protein 10 | -5.15 | down | 4.10 | up | -2.40 | down |
| Sobic.003G106500 | turquoise | NA | -1.12 | down | -1.68 | down | -2.38 | down |
| Sobic.010G210600 | turquoise | Chaperone DnaJ-domain superfamily protein | -4.27 | down | 3.24 | up | -2.38 | down |
| Sobic.005G091200 | pink | NA | 4.12 | up | 2.54 | up | 2.29 | up |
| Sobic.003G321101 | red | nuclear transport factor 2B | -3.05 | down | 4.40 | up | -2.24 | down |
| Sobic.002G345850 | yellow | NA | 3.16 | up | -2.95 | down | -2.24 | down |
| Sobic.006G179500 | red | Protein kinase superfamily protein | -1.75 | down | 3.36 | up | -2.23 | down |
| Sobic.006G024500 | turquoise | Cystathionine beta-synthase (CBS) family protein | -2.31 | down | -1.07 | down | -2.21 | down |
| Sobic.010G039200 | turquoise | O-acetylserine (thiol) lyase B | -1.69 | down | -1.30 | down | -2.21 | down |
| Sobic.001G276100 | turquoise | peptide transporter 2 | -4.82 | down | -1.67 | down | -2.20 | down |
| Sobic.010G057633 | turquoise | HSP20-like chaperones superfamily protein | -2.64 | down | 3.02 | up | -2.17 | down |
| Sobic.006G202300 | blue | Homeodomain-like superfamily protein | 3.55 | up | 1.52 | up | 2.15 | up |
| Sobic.010G028000 | turquoise | ribosome-binding factor A family protein | -2.36 | down | -1.88 | down | -2.15 | down |
| Sobic.007G144900 | green | glutamine dumper 3 | 5.42 | up | -2.72 | down | 2.14 | up |
| Sobic.009G238100 | turquoise | Ubiquitin carboxyl-terminal hydrolase  family protein | -3.26 | down | -2.34 | down | -2.06 | down |
| Sobic.004G302150 | turquoise | NA | -1.70 | down | -1.93 | down | -2.05 | down |
| Sobic.006G020200 | red | HXXXD-type acyl-transferase family protein | -1.97 | down | 3.67 | up | -2.02 | down |
| Sobic.010G248301 | violet | choline monooxygenase, putative (CMO-like) | 1.40 | up | -2.43 | down | 1.98 | up |
| Sobic.007G004850 | green | NA | 5.79 | up | -2.27 | down | 1.92 | up |
| Sobic.001G416300 | turquoise | NA | -1.05 | down | -1.02 | down | -1.90 | down |
| Sobic.001G195100 | turquoise | cytochrome P450, family 87,  subfamily A, polypeptide 6 | -3.22 | down | 1.80 | up | -1.88 | down |
| Sobic.006G014700 | red | cinnamyl alcohol dehydrogenase 9 | -1.62 | down | 3.55 | up | -1.85 | down |
| Sobic.005G062900 | yellow | NB-ARC domain-containing  disease resistance protein | 1.04 | up | -1.70 | down | -1.82 | down |
| Sobic.006G057866 | darkgrey | pseudo-response regulator 7 | -2.34 | down | 2.02 | up | 1.81 | up |
| Sobic.009G052700 | turquoise | NA | -1.10 | down | -2.13 | down | -1.80 | down |
| Sobic.003G097701 | violet | NA | 5.36 | up | -1.55 | down | 1.78 | up |
| Sobic.004G032400 | blue | L-aspartate oxidase | 3.55 | up | 3.05 | up | 1.78 | up |
| Sobic.007G047400 | blue | Homeodomain-like superfamily protein | 7.38 | up | 1.67 | up | 1.77 | up |
| Sobic.004G279300 | blue | Homeodomain-like superfamily protein | 2.76 | up | 4.44 | up | 1.75 | up |
| Sobic.009G163900 | black | heat shock protein 70 | 3.23 | up | 2.43 | up | 1.72 | up |
| Sobic.008G180800 | blue | PHYTOENE SYNTHASE | 4.45 | up | 2.50 | up | 1.72 | up |
| Sobic.002G408100 | black | sodium hydrogen exchanger 2 | 3.94 | up | -1.26 | down | 1.70 | up |
| Sobic.009G154600 | turquoise | Glycosyl hydrolase superfamily protein | -2.10 | down | -2.23 | down | -1.69 | down |
| Sobic.007G077200 | green | erf domain protein 9 | 2.52 | up | -2.05 | down | 1.68 | up |
| Sobic.003G058300 | yellow | NA | 3.80 | up | -1.91 | down | 1.66 | up |
| Sobic.009G234200 | turquoise | 16S rRNA processing protein RimM family | -1.24 | down | -1.75 | down | -1.66 | down |
| Sobic.001G161400 | turquoise | Primosome PriB/single-strand DNA-binding | -2.08 | down | -1.79 | down | -1.65 | down |
| Sobic.010G183400 | turquoise | overexpressor of cationic peroxidase 3 | -1.53 | down | -1.08 | down | -1.65 | down |
| Sobic.002G174000 | yellow | pleiotropic drug resistance 12 | 1.79 | up | -2.02 | down | 1.64 | up |
| Sobic.008G001000 | yellow | phototropin 1 | 1.74 | up | -2.82 | down | 1.63 | up |
| Sobic.003G207900 | turquoise | Transmembrane amino acid transporter  family protein | -4.34 | down | 3.83 | up | -1.60 | down |
| Sobic.005G167800 | blue | NA | 2.01 | up | 2.49 | up | 1.59 | up |
| Sobic.002G386400 | turquoise | chloroplast RNA-binding protein 29 | -1.79 | down | -1.86 | down | -1.56 | down |
| Sobic.001G078200 | turquoise | maternal effect embryo arrest 14 | -1.60 | down | -3.40 | down | 1.50 | up |
| Sobic.002G300400 | turquoise | ACT-like protein tyrosine kinase  family protein | -2.05 | down | -2.37 | down | 1.47 | up |
| Sobic.003G209900 | yellow | photosystem II light harvesting  complex gene B1B2 | 4.16 | up | -3.63 | down | 1.46 | up |
| Sobic.001G410300 | turquoise | NA | -1.66 | down | 1.04 | up | -1.46 | down |
| Sobic.003G344200 | yellow | Plant protein of unknown function (DUF828) | 2.82 | up | -1.62 | down | -1.43 | down |
| Sobic.010G054600 | turquoise | ribonucleotide reductase 1 | -2.38 | down | -1.16 | down | 1.42 | up |
| Sobic.005G169600 | turquoise | CRM family member 3A | -2.40 | down | -1.87 | down | -1.40 | down |
| Sobic.003G221200 | turquoise | CTP synthase family protein | -1.20 | down | -1.27 | down | 1.34 | up |
| Sobic.005G186500 | turquoise | serine carboxypeptidase-like 27 | -3.46 | down | 3.51 | up | -1.32 | down |
| Sobic.007G190200 | white | purple acid phosphatase 27 | -2.00 | down | 2.32 | up | -1.30 | down |
| Sobic.003G076900 | green | sulfoquinovosyldiacylglycerol 2 | 2.74 | up | -2.05 | down | 1.25 | up |
| Sobic.006G176100 | turquoise | NA | -1.33 | down | -1.40 | down | -1.24 | down |
| Sobic.002G226100 | turquoise | protein disulfide isomerases | -1.85 | down | -1.34 | down | -1.24 | down |
| Sobic.008G165500 | turquoise | NA | 1.96 | up | -1.52 | down | 1.22 | up |
| Sobic.009G173600 | turquoise | ATPases;nucleotide binding;ATP binding;nucleoside-triphosphatases;  transcription factor binding | -2.25 | down | -1.20 | down | 1.21 | up |
| Sobic.003G428300 | greenyellow | response regulator 9 | -2.17 | down | 1.64 | up | 1.20 | up |
| Sobic.010G090301 | turquoise | Protein of unknown function (DUF581) | -1.45 | down | 1.25 | up | -1.20 | down |
| Sobic.001G377000 | violet | Dormancy/auxin associated family protein | 1.60 | up | -1.55 | down | 1.16 | up |
| Sobic.003G296600 | darkred | NA | 1.69 | up | -1.14 | down | 1.12 | up |
| Sobic.005G200300 | blue | Tyrosine transaminase family protein | 2.28 | up | 1.14 | up | 1.11 | up |
| Sobic.006G258000 | turquoise | regulatory protein RecX family protein | -1.61 | down | 1.15 | up | -1.09 | down |
| Sobic.005G132400 | green | Glycosyl hydrolase family 38 protein | 1.16 | up | -1.35 | down | 1.07 | up |
| Sobic.004G071800 | yellow | NA | 1.29 | up | -2.60 | down | 1.07 | up |
| Sobic.004G247700 | darkturquoise | NA | -2.87 | down | 3.19 | up | -1.04 | down |
| Sobic.006G111600 | turquoise | cell division protein ftsH, putative | -1.41 | down | -1.22 | down | -1.04 | down |
| Sobic.004G225700 | turquoise | RNI-like superfamily protein | -3.25 | down | 2.47 | up | -1.01 | down |
